# Bone Multicellular Unit on a Chip (BMU-Chip) Subjected to Cyclic Mechanical Loading

**DOI:** 10.1021/acsbiomaterials.5c01798

**Published:** 2026-03-11

**Authors:** Anna-Blessing Merife, Michael P. Seitz, Angelika Polshikova, Ujjwal Aryal, Zachary J. Geffert, Era Jain, Jason Horton, Paola Divieti Pajevic, Pranav Soman

**Affiliations:** † Department of Chemical and Biomedical Engineering, 2029 Syracuse University,Syracuse, New York 13244, United States; ‡ Department of Translational Dental Medicine, Goldman School of Dental Medicine, 1846 Boston University, Boston, Massachusetts 02215, United States; § Department of Neuroscience and Physiology, Alan and Marlene Norton College of Medicine, 12302 SUNY Upstate Medical University, Syracuse, New York 13210, United States

**Keywords:** cyclic mechanical stimuli, osteocytes, osteoblasts, osteoclasts, coculture, bone multicellular unit, in vitro model, microfluidic device

## Abstract

The skeleton undergoes continuous remodeling to maintain its structural integrity. The basic unit of bone remodeling is the Bone Multicellular Unit (BMU), a highly organized complex of osteocytes, osteoblasts, and osteoclasts that remodels skeletal microarchitecture to adapt to the mechanical demands placed upon the bone. Here, we describe the design and development of the BMU-chip microfluidic platform for the longitudinal investigation of complex interactions between different cell types and their extracellular matrix in response to cyclic mechanical loading. Three-chambered polydimethylsiloxane (PDMS) chips are fabricated using a combination of 3D-printed master molds and soft lithography compatible with real-time, time-lapse, and confocal microscopy methods. The chip is then populated with murine osteocytes (OCY454) suspended in a collagen gel matrix, allowing the self-assembly of three-dimensional networks. Murine preosteoblastic (MC3T3-E1.4) and preosteoclastic (Raw264.7) cells are introduced to a parallel chamber of the device and induced to differentiate in situ, forming the cellular and matrix components of the BMU-chip, which are evaluated in mono- and coculture configurations. Pulsed Unidirectional Fluid Flow Stimuli (PUFFS) are then applied to the osteocyte network via the third parallel chamber. Over a period of up to 31 days of PUFFS stimulation, cells in the devices demonstrated excellent cell viability and lacunocanalicular morphology and expressed cell-specific phenotypic markers as assessed by gene expression and immunofluorescence studies. Throughout the experiment, live-cell fluorescence microscopy was used to study PUFFS-evoked Ca2+ signal propagation through the osteocyte network. These results suggest that the BMU model will be a useful experimental platform for studying key aspects of skeletal mechanoadaptation that cannot be feasibly studied by contemporary methods.

## Graphical abstract

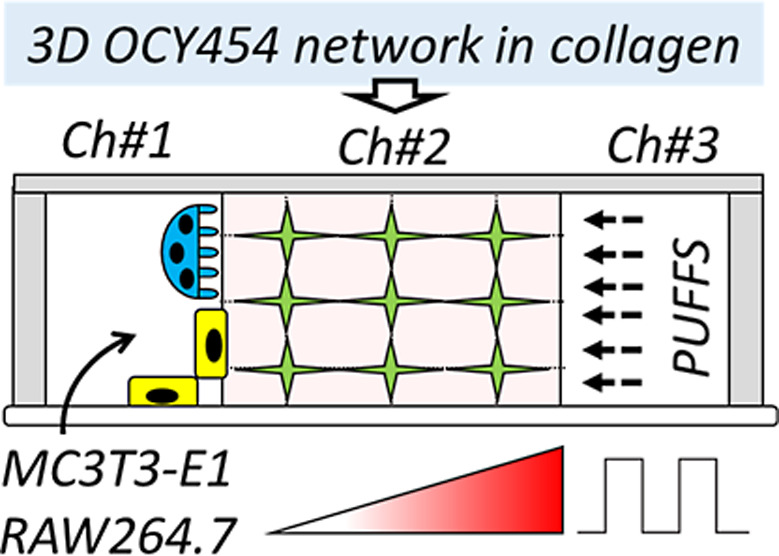

## Introduction

Osteocytes are the primary mechanosensory cells in bone, crucial for detecting mechanical loads and coordinating bone modeling and remodeling activities.^1–6^ Individual osteocytes in living bone are entombed in a mineralized collagen-rich matrix. The lacunocanalicular system is an extensive intercellular network through the bone matrix connecting osteocytes to each other as well as to their effector cells. Upon loading, osteocytes sense matrix deformation and interstitial fluid flow-through and rapidly transmit biochemical and ionic signals across gap junctions, which rapidly propagate in a wave-like fashion across the lacunocanalicular network. This mechanism is critical to direct the spatial and temporal activity of bone-forming osteoblasts and bone-resorbing osteoclasts to adapt to the stresses placed on the bone^7^ and maintain skeletal homeostasis.^8–10^ Defects in osteocyte mechanotransduction are implicated in several skeletal diseases, including osteoporosis, osteoarthritis, osteotropic metastases,^11–13^ congenital bone diseases,^14^ fracture healing,^15^ and aseptic implant loosening.^16^ In order to develop and validate new therapeutic interventions that promote skeletal health, it is important to understand how transient mechanically evoked signals drive durable mechanoadaptive remodeling within the Bone Multicellular Unit (BMU) consisting of osteocytes, osteoblasts, and osteoclasts.^11–22^

Most research on osteocyte mechanobiology has focused on mechanically evoked calcium (Ca^2+^) transients.^23–27^ Direct visualization of Ca^2+^ signaling has traditionally involved imaging of live bone explants or animal models subjected to brief (<24h) bouts of mechanical loading.^28–31^ This requires extensive instrumentation, invasive surgical preparation, and a complex apparatus for loading and imaging, limiting the capacity to examine multiple samples in parallel and over long time scales. These studies are also costly, ethically questionable, and constrained by short observation periods.^32–34^ Furthermore, the opaque nature of mineralized bone prevents penetration of light at wavelengths necessary to visualize fluorescent Ca2+ probes, confounding the ability to resolve signaling beyond the bone surface. Likewise, conventional in vitro models have significant drawbacks that limit their physiologic relevance. A wide range of in vitro two-dimensional (2D) culture models have been developed that largely rely on immortalized murine osteocytes^35–42^ that in isolation alone cannot replicate the three-dimensional architecture and cellular complexity of the BMU. Simple osteocyte monocultures have been adapted for coculture models to study the interactions with osteoblastic and/or osteoclastic cells.^43–53^ Fluid shear models involve stimulation by passing a culture medium over sheets of cells, while nanoindentation models have been used to observe single-cell responses. While widely used, these models notably fail to replicate the three-dimensional lacunocanalicular system observed in vivo, which is critically important to account for in models of mechanotransduction. To mimic 3D osteocyte networks, osteocytes in the presence of mineralized 3D scaffolds, hydroxyapatite particles, and hydrogels have also been developed;^47,54^ however, as with native bones, the opacity of such models limits the observable depth available for real-time visualization of signaling activity. Finally, these systems are not generally compatible with time-lapse imaging approaches necessary for execution of longitudinal experiments, at a time scale necessary to observe both mechanically evoked transient signals like Ca2+, that orchestrate the latent yet durable remodeling cycles of bone formation and resorption that constitute the mechanoadaptative response to loading.

We recently reported a relatively simple microfluidic model that enabled real-time visualization of Ca2+ responses across a self-assembled 3D network of collagen-suspended osteocytes in response to Pulsatile Unidirectional Fluid Flow Stimuli (PUFFS).^55^ As a monoculture model, other cells relevant to the BMU were not included, thus limiting its utility for studies aimed at understanding how mechanically loaded 3D osteocytes coordinate the mechanoadaptive responses of osteoblastic and osteoclastic effector cells. To resolve this limitation, we sought to design and validate a novel BMU chip that integrates the following capabilities: (a) an ability to apply defined mechanical stimuli in the form of PUFFS to 3D osteocyte networks (OCY454s, OCY) for more than 30 days, (b) an ability to coculture osteoblasts (MC3T3-E1, OB) and osteoclasts (RAW264.7, OC) in selected chambers at user-defined time-points, and (c) an ability to detect real-time signals (Ca^2+^ signals) and longer-term outcomes (viability, morphology, protein and gene expression) using standard assessment methods (time-lapse imaging, immunostaining, qPCR).

## Results

### Process Workflow for Performing Mono-/Cocultures within Three-Chambered Microfluidic Chips

To fabricate three-chambered PDMS chips, master molds were printed using projection stereolithography (PSLA), followed by replica casting, thermal curing, and plasma bonding to glass coverslips (22 mm × 22 mm)^55^ (Figure 1A–C). Fabricated chips consist of a central chamber (Ch#2) (850 μm wide, to house OCY454s encapsulated within 3D collagen gels) flanked by two side chambers (∼500 μm wide), and each chamber has inlet and outlet ports (2 mm diameter). The height of all the chambers is 250 μm, while the overall thickness of the chip is ∼5 mm. In this work, chamber 1 (Ch#1) was used to seed model effector cells (OB, OC) at defined time points, while chamber 3 (Ch#3) was used to apply cyclic loading in the form of daily PUFFS (Figure 1D). Chip surfaces were coated with a polydopamine (PDA) solution, and OCY cells and a collagen solution were pipetted in Ch#2, followed by gelation at 37 °C for 30 min. During the gelation process, the micropost array ensures that the solution is confined to Ch#2 and does not leak into the side chambers. Postgelation, the side chambers were filled with media and cultured under standard conditions.

**Figure 1:**
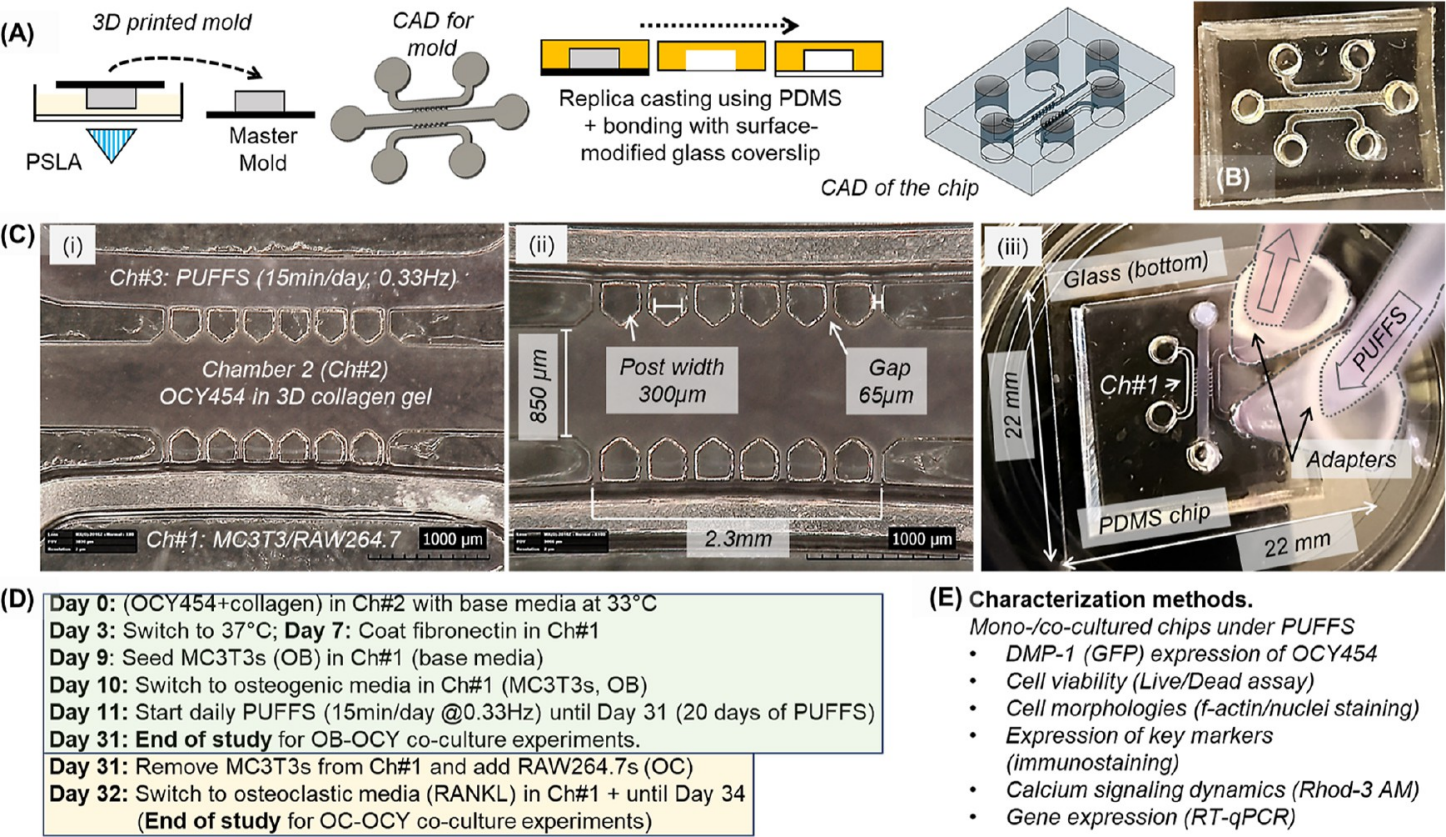
(A) Process flow showing the design and fabrication process to make final PDMS chips. (B) Representative photograph of the final PDMS chip (top view) bonded to a glass coverslip. (C) (i) Top view of the chip showing three chambers separated by an array of microposts: OCY in 3D collagen gel will be incorporated within Ch#2, and PUFFS will be applied from Ch#3, while Ch#1 is reserved for seeding and culturing OBs or OCs at user-defined time-points, (ii) various dimensions of the chip, and (iii) picture showing application of PUFFS in Ch#3 via tubing connected to peristaltic pumps. (D) Sequence of events for modeling OB-OCY and OC-OCY coculture systems up to 34 days. (E) Brief overview of the various characterization methods used for this study.

Figure S1 shows the setup used to apply PUFFS to 4 independent chips; this consists of peristaltic pumps, frequency monitor, controller, tubing, and adapters. Details can be found in our previous work.^55^ In this work, PUFFS (0.33 Hz, 15 min/daily) was applied to Ch#3 of the chips at specific time ranges based on the experimental design. During PUFFS, the medium was recirculated via inlet/outlet tubing inserted into the ports in Ch#3. A 3D printed stabilizer was used to mitigate unwarranted movement from the connected chips during imaging and generate reproducible calcium signal recordings. To test the influence of PUFFS on the collagen gel, fluorescent beads (1 μm diameter) were gelled within collagen (4 mg/mL) within chamber no. 2 of the chip and exposed to PUFFS (0.33 Hz). Unlike our previous work with 2.5 mg/mL collagen, which showed significant deformation,^55^ we did not observe any bead movement following PUFFS or any visible damage to the collagen gel for the duration of the study (Day 34).

A modular study design was adopted to enable the application of PUFFS to 3D osteocyte networks cultured alongside OBs or OCs and thus capture the essential features of BMU (Figure 1D,E). For coculture experiments, Ch#1 is coated with fibronectin (Days 7 and 8) before seeding a solution of OBs (3 × 10^4^ cells/mL) in Ch#1 on Day 9. PUFFS were applied from Day 11 to 34 in Ch#3, while an osteogenic differentiating medium was used to culture OBs in Ch#1. For sequential coculture experiments, cells from Ch#1 on Day 31 are washed with a dilute trypsin solution and medium before seeding OCs (2 × 10^5^ cells/ml) and culturing them using an osteoclastic differentiating medium for 3 more days. For each condition tested, control groups included a static control (no PUFFS in Ch#3) and monocultured chips. At defined time points and for various conditions, cell viability and morphologies, protein and gene expressions, and calcium signaling response were characterized.

### Osteocyte Maturation and Viability Are Supported by Extended Culture in BMU-Chip Devices and Enhanced by PUFFS Stimulation

Dentin matrix protein 1 (DMP1) is an acidic glycoprotein expressed in the bone and tooth extracellular matrix that coordinates nucleation of hydroxyapatite along type I collagen fibers. DMP1 is expressed at low to undetectable levels in proliferating osteoprogenitor and osteoid-secreting osteoblasts; expression gradually increases as the cell ceases proliferation and becomes entombed in its matrix. Expression of DMP1 peaks as the cell begins mineralizing its pericellular lacuna, signifying maturation into a terminally differentiated osteocyte. To determine whether our model supports osteocyte maturation and evaluate how PUFFS stimulation may alter this dynamic process, we selected the murine osteocyte-like cell line OCY454. These cells are conditionally immortalized by a temperature-sensitive variant of the SV40 Large T antigen and express a Green fluorescent protein (GFP) reporter controlled by the dentin matrix protein 1 (DMP1) transcriptional promoter. These cells resemble proliferating osteoblasts when maintained at 33 °C, but culture at a semipermissive temperature of 37 °C, and inactivate the SV40 large T antigen, which triggers mitotic arrest and initiates maturation into terminally differentiated osteocytes, marked by expression of the DMP1-GFP reporter.^56^ Furthermore, these cells have been shown to support differentiation of both osteoblasts and osteoclasts and are further modulated by mechanical stimulation.^57^

Two days after seeding with OCY454 suspended in collagen, our devices were transferred to the growth-restricting temperature condition (37 °C) to initiate arrest and osteocytic maturation and subjected to PUFFS (0.5 Hz, 15 min/day) or static treatment. Live–dead staining demonstrated that cell viability was preserved throughout the observation period in both conditions (Figure 2C, Di). In both conditions, some of the OCY were observed to migrate out of the collagen matrix in Ch2, along the glass and PDMS surfaces of both Ch1 and Ch3. On day 11, and at 4 day intervals thereafter through day 31, expression of the DMP1-GFP transgene was evaluated by fluorescence and phase-contrast microscopy (Figures 2B,Dii and S2). Interestingly, the cells that migrated into Ch1 or Ch3 showed very low levels of DMP1-GFP reporter expression on day 31. This may indicate that departure from the 3D environment could suspend progression along the osteoblast-to-osteocyte continuum, in contrast to those remaining in the 3D environment of Ch2 (Figures 2Bi,S2B). While osteoprogenitors and osteoblasts are capable of migration along the bone surface, we are not aware of any study demonstrating bone-embedded osteocytes migrating out of their lacunae and onto the bone surface. Previous studies with this cell line described culturing at 37 °C as “semipermissive” conditions, sufficient to induce mitotic arrest and osteocytic maturation in the vast majority of cells.^56,58^ However, the tsA58 variant of SV40 large T requires culture at 39 °C for complete inactivation,^59^ suggesting that the migratory cells lacking DMP1-GFP were able to escape selection and persist in a proliferative osteoblast-like state due to incomplete inactivation.

**Figure 2:**
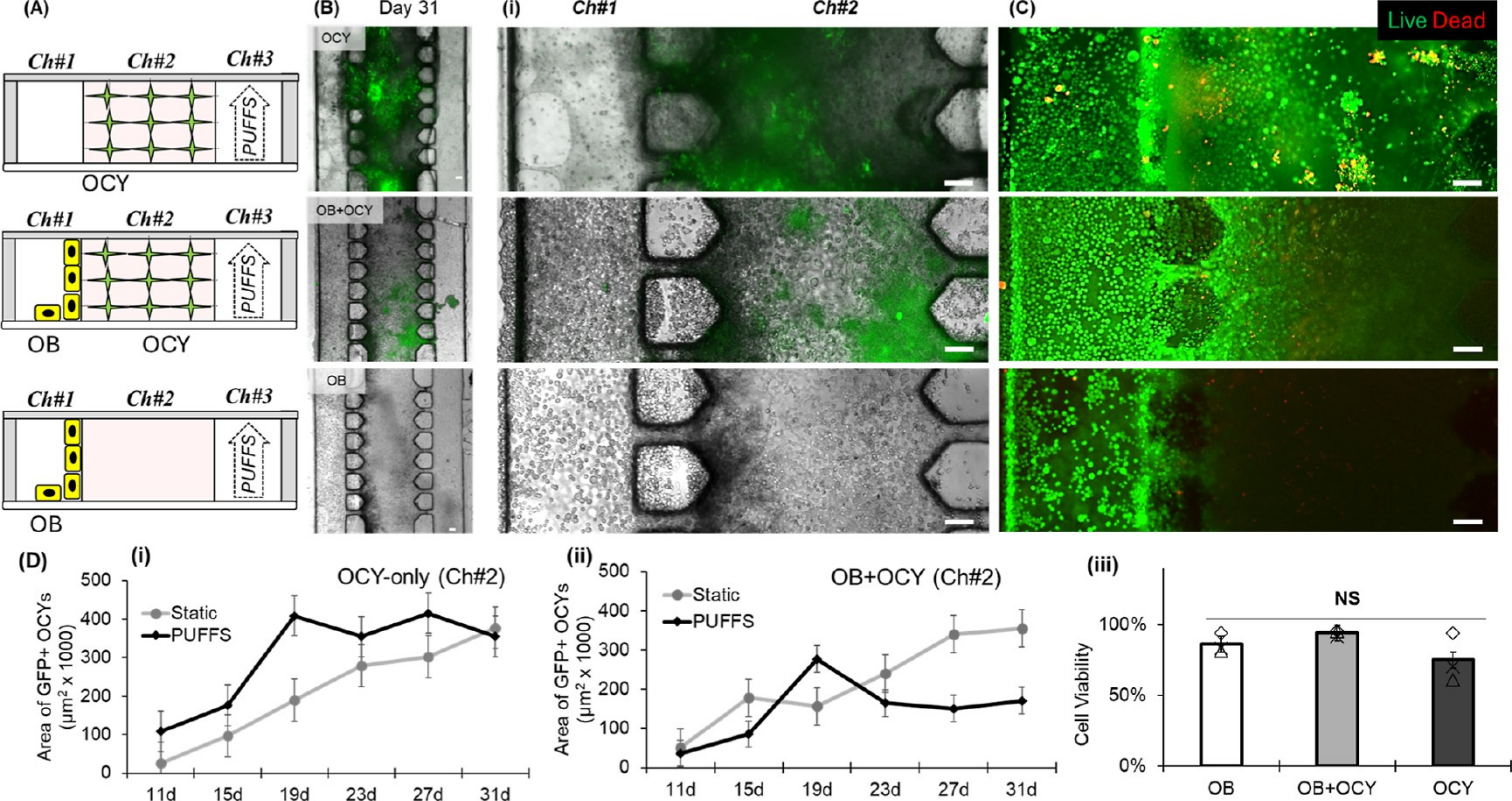
(A) Side-view schematics of mono- and cocultures in a three-chambered microfluidic chip. (B–Bi) Composite bright-field and fluorescence day 31 images showing OCY expressing GFP in Ch#2 of the chip. Scale bar = 100 μm. (C) Viability of mono- and co cultures (green = live; red = dead). Scale bar = 100 μm. (D) (i,ii) Changes in GFP + OCYs in Ch#2 as a function of culture duration and (iii) cell viability between mono- and co-cultured chips. Data analyzed from 3 independent chips.

Under static conditions, DMP1 expression gradually increased through the observation period to a peak on day 31, indicating that our BMU-on-chip approach supports osteocytic differentiation. Chips exposed to daily PUFFS stimulation showed higher initial levels of DMP1 expression, reaching a similar peak expression on day 19 and remaining approximately constant for the duration of the experiment thereafter (Figures 2Di and S2). This suggests that PUFFS stimulation accelerates osteocyte maturation, consistent with findings by others using a variety of 2D, 3D, and in vivo model systems, demonstrating mechanical stimulation increases expression of DMP1 and other markers of osteocyte maturation at earlier time points^57,60–62^ (Supporting Information Table S3) and hastens development of morphological features of osteocyte maturation including lacunar mineralization, lacuna-canalicular connectivity, and gap junctions that are critical for osteocyte function as mechanosensors and orchestrators of mechanoadaptation.^63–66^

### Coculture with Osteoblasts Attenuates Maturation of Osteocytes When Subjected to PUFFS

The overarching goal of our study was to advance our experimental platform to more closely replicate the in vivo BMU and intercellular communication in response to mechanical stimulation. Therefore, we devised experiments to evaluate incorporating osteoblast-like MC3T3-E1.4 into our BMU-chip platform in monoculture (OB-only) or in co-culture with collagen suspended on the adenosine-free OCY454 cells. In brief, devices were assembled, and Ch2 was loaded either with cell-free collagen or with OCY454 suspended in collagen and subjected to daily bouts of PUFFS or static conditions as described above. On day 9, MC3T3-E1.4 cells were seeded in Ch1, exposed to osteogenic induction media on day 10, and observed at 4 day intervals for another 20 days. In the presence or absence of osteocytes, the OB in Ch1 retained high cell viability (Figure 2C,D1). In monoculture, OB remained in Ch1, though some appeared to invade Ch2, appearing along the glass and PDMS surfaces as well as through the collagen matrix (Figure S3) under both static and PUFFS conditions.

When cocultured with osteoblasts under static conditions, DMP1 expression followed a similar trajectory as that of OCY in monoculture, indicating that the mere presence of OB in this system does not alter the rate of maturation of OCY maturation. Under PUFFS conditions, however, coculture with OB appeared to attenuate the OCY maturation, and DMP1-GFP reporter expression peaked on day 19, before gradually receding toward baseline levels (Figure 2C,Diii,S). This finding appears to align with that of Skottke et al., who reported significantly lower DMP1 expression in a primary human osteocyte in vitro coculture system with primary human osteoblasts,^51^ suggesting that experimental conditions, direct cell–cell contact, and cell-type differences may contribute to this effect.

### Cell Morphology under Mono- and Coculture Conditions

Cell morphology was assessed by staining the cell nucleus (DAPI; blue) and cytoskeleton (f-actin; green) (Figure 3 A–C). We tested whether cell morphologies were affected by PUFFS or coculture conditions. Since the total height of the collagen was ∼250 μm, we captured *Z*-stack images through the collagen compartment. For analysis, image slices were sampled at different *z*-depths: at the bottom surface (z ∼ 0), at z ∼ 70 μm from the bottom, and at a *z*-plane close to the top PDMS surface (∼200 μm from the bottom) (Figure S3 and Video S1).

**Figure 3:**
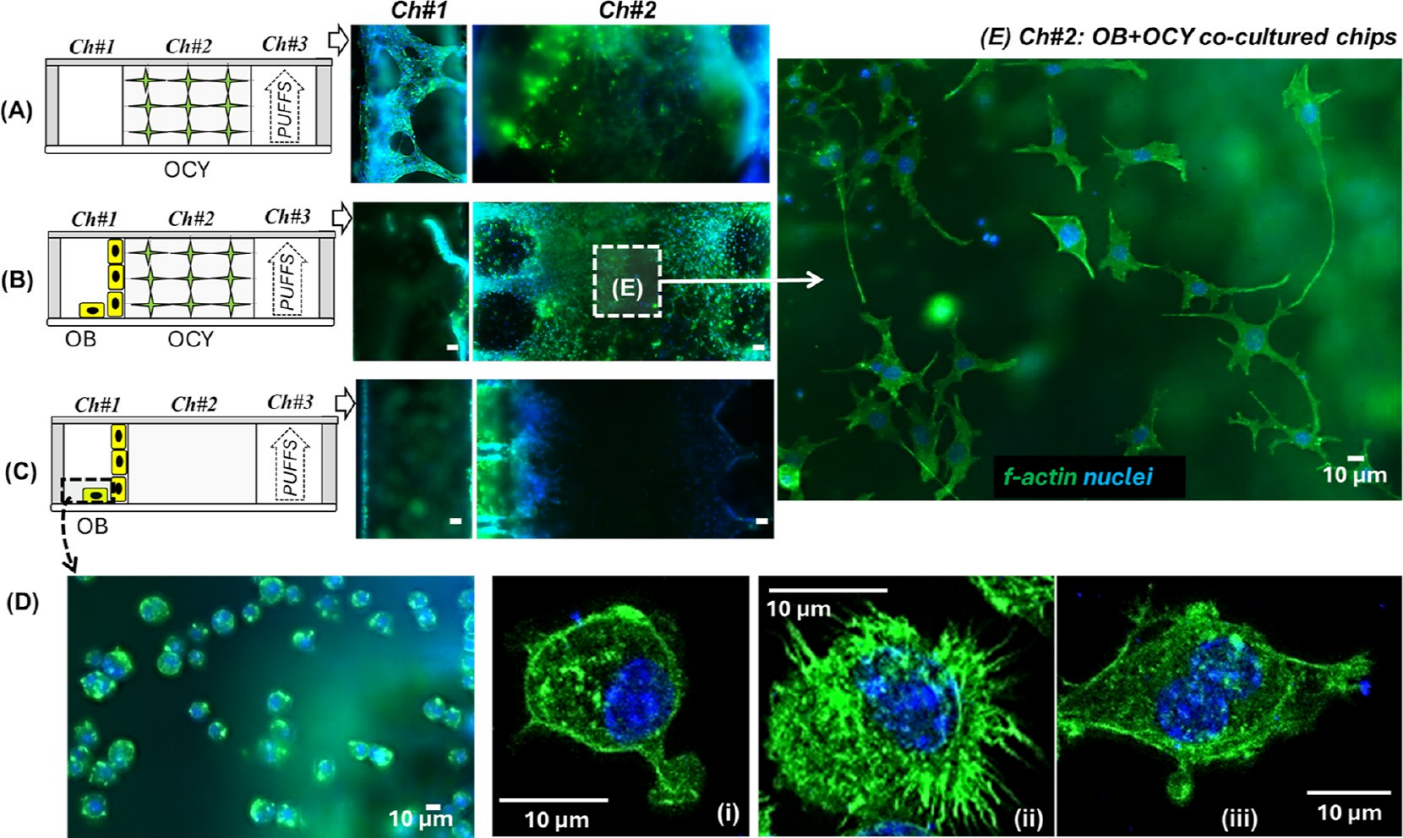
(A–C) Side-view schematics of mono- and co-cultures in a three-chambered microfluidic chip and representative fluorescence images from Ch#1 and Ch#2 midsection (∼70 μm in the *z*-plane) of mono- and co-culture chips. Scale bar = 50 μm. (D) Morphology of OBs in Ch#1. Scale bar = 10 μm. (E) Representative images from the OB + OCY chip showing dendritic connections from the center of Ch#2 (dashed box).

Throughout the image stacks, we observed thin sheets of cells in both the OCY-only and OB + OCY chips subjected to PUFFS, while these structures were not observed in the static control chips. Mechanical loading has been shown to activate physical and biochemical cues that reinforce osteocyte connectivity and matrix/cytoskeletal interactions.^67,68^ We suspect that this likely reflects the strain-deformation fields created by PUFFS, characterized in previous work.^69^ This organization may be initiated during the early network self-assembly phase, where cells orient their cell body and extend canalicular processes along these strain fields. Over time, the cell sheets may coalesce as long as these fields have some “favorable” intercellular connections reinforced by robust cytoskeletal structures, while cells not receiving the mechanical stimulus may withdraw canalicular processes extending between cell sheets. Based on monoculture or coculture conditions, we observed some variations in cell morphology. For instance, for OB-only chips, OB cells adhere on the bottom (on glass) and on the top (on PDMS) and migrate into the collagen in Ch#2 (Figure 3Di–iii). Under coculture conditions (OB + OCY), a similar dense network was generated, but it invaded Ch#1 to a lesser extent as compared to the OCY-only chip. In some locations, we observed dendritic-like extensions within OCYs in Ch#2 (Figure 3E).

### Sequential Sample Harvesting Approach Allows Parallel Evaluation of Gene Expression of Distinct Cell Populations under Mono- and Coculture Conditions

A key objective in designing our experimental platform was the ability to recover samples from each channel to perform a parallel analysis of gene expression. To accomplish this, we devised a sequential harvest approach as described in Materials and Methods, followed by RT-qPCR for a panel of osteoblast and osteocyte marker genes (Figure 4 and Supporting Information Table S1). As proof-of-concept experiments aimed at validating cell phenotype after 31 days in the device under daily PUFFS stimulation, data is presented as the expression level, relative to the geometric mean of a panel of housekeeping genes. ANOVA was used to assess the significance of the difference in expression between each population.

**Figure 4:**
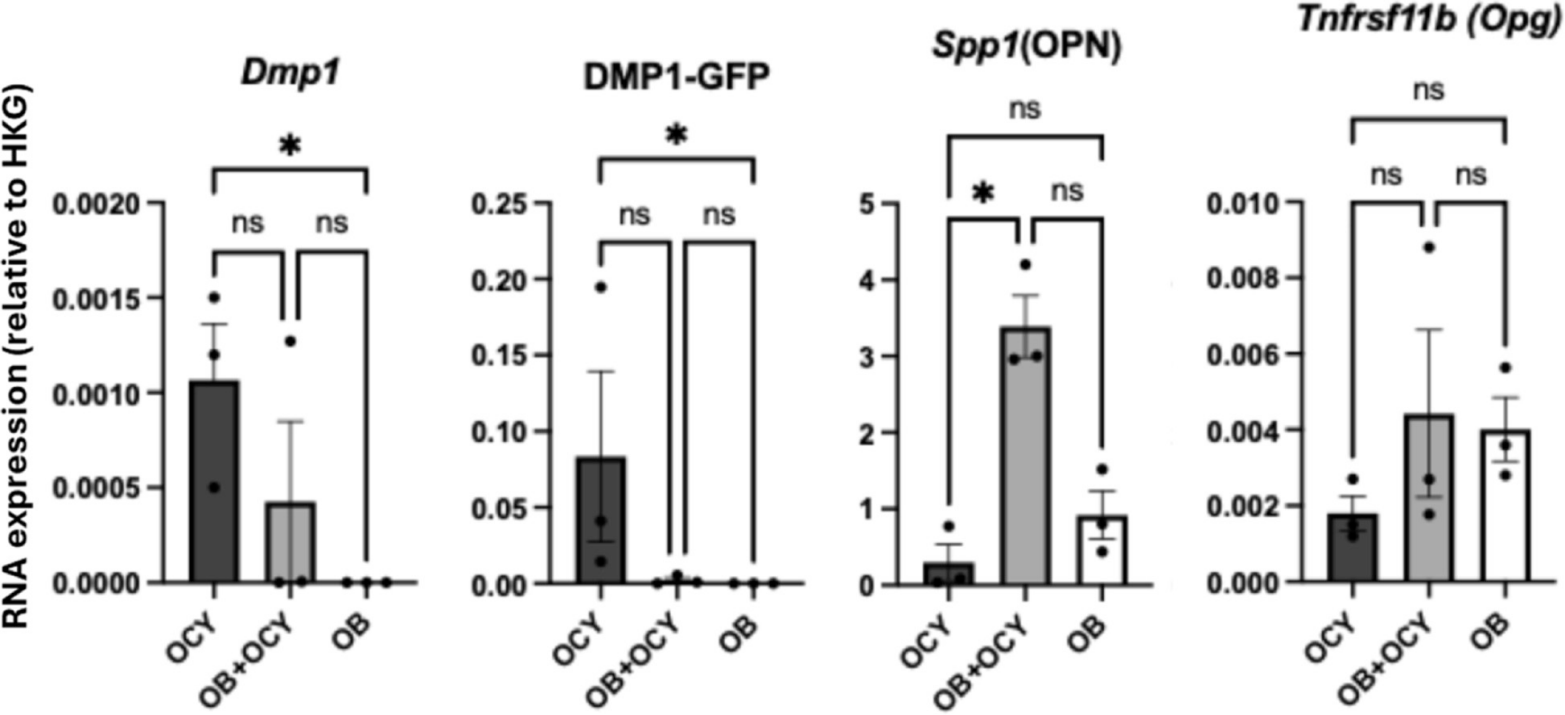
Expression of phenotypic marker transcripts by RT-qPCR.

As noted with respect to our choice of the OCY454 cell line, *Dmp1* is a matrix SIBLING protein that promotes osteocyte maturation, maintains the lacunocanalicular network, and contributes to phosphate homeostasis partly via regulation of Fgf23.^70^ Expression of DMP1 rises from low/absent in early osteoblasts to moderate in late osteoblasts/preosteocytes and high in mature osteocytes and is enhanced by mechanical loading. In our experiments, DMP1, as well as the DMP1-GFP reporter, was not detected in OB monocultures, which are highly expressed in the osteocyte monoculture. DMP1 was also detected in the cocultures, likely reflecting the migration of the OCY454 cells out of the collagen gel in Ch2 into Ch1. Transient exposure of osteocytes to dexamethasone can increase DMP1 exposure, and chronic exposure can induce osteocytic stress and apoptosis.^71,72^

Both osteocytes and osteoblasts express Osteopontin (*Spp1*), a noncollagenous matrix protein that promotes osteoclast attachment to the bone matrix via integrin binding RGD motifs. Various mechanical stimuli (tensile strain, compression, fluid shear) upregulate Opn/SPP1, particularly at sites of high strain and microdamage.^73–75^ In our study, *Spp1* was expressed in both OB and Ocy monocultures and was highest in the coculture condition.

Osteoprotegerin (*Tnfrsf11b*) encodes a decoy receptor for RANKL that inhibits osteoclastogenesis and, thereby, modulates bone remodeling. It is expressed at moderate to high levels in osteoblasts and osteoblastic stromal cells and at low to moderate levels in osteocytes. Mechanical loading generally shifts the Opg/Rankl balance toward bone formation. In our experiment, OPG was detected at similar levels in all culture configurations. Rank-L expression was not assayed in this study.

While a number of additional osteoblast and osteocyte marker genes were assayed in this study (Supporting Information Figure S8), we acknowledge that inclusion of dexamethasone in the osteogenic differentiation medium is a major confounder in these experiments because it directly impacts expression of many osteogenic genes.^71,76^ Nonetheless, these results reinforce that this experimental platform is compatible with gene expression by isolating RNA from distinct cell populations in each channel of the chip, allowing parallel evaluation from single experiments.

### Immunohistochemical Expression of Key Markers

An additional functionality sought in the design of the BMU-Chip was the ability to use immunohistochemical methods to probe the expression of marker proteins by cells located throughout the device. We stained for key proteins such as (i) GFP to identify OCYs within the chips and (ii) Runt-related transcription factor 2 (Runx2) activity, a marker of early osteoblast differentiation (Figure 5). As expected, OCY-only chips express high levels of GFP in Ch#2 as compared to cocultured or OB-only chips. In OB monocultures, Runx2 expression was visualized in both Ch1 and by cells invading Ch2, while GFP was undetected. A similar pattern of Runx2 staining was observed in cocultures, though fewer OB were observed to migrate into Ch2, consistent with our earlier findings, as shown in Figure 2. In both OCy monoculture and OB + OCy cocultures, GFP staining was primarily localized to Ch2, though some staining was observed in Ch1, presumably marking cells that had migrated out of the 3D environment, again consistent with our earlier findings, as shown in Figure 2. Interestingly, some cells in OCy monoculture chips that migrated into Ch1 displayed Runx2 staining, which may suggest they had not been induced to osteocytic differentiation or alternatively had dedifferentiated to a more primitive osteoblastic state. Importantly, however, Runx2 and GFP signals did not colocalize in individual cells in any experiment, indicating high stringency of the immunostaining procedure.

**Figure 5:**
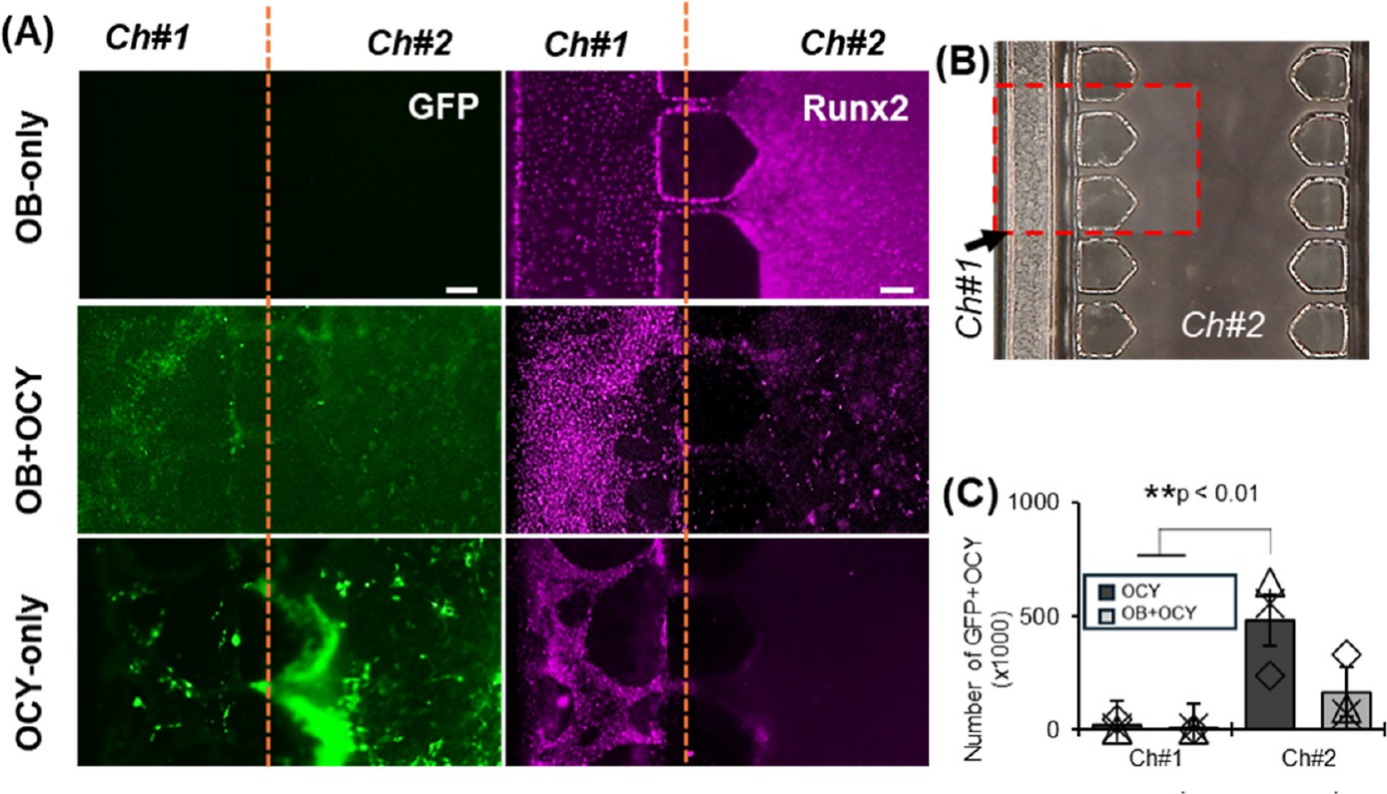
(A) Representative fluorescent images showing the expression of GFP (green) and Runx2 (pink) for monoculture (OB-only and OCY-only) and coculture (OB + OCY). Scale bar = 100 μm. (B) Red dashed box showing ROI within the chip. (C) Plots showing the area of GFP expression from Ocy454 from Ch#1 and Ch#2.

### Real-Time Monitoring of Calcium Signaling

Both monocultured and cocultured chips were subject to daily PUFFS (15 min/day at 0.33 Hz), and on Day 31, time-lapse confocal microscopy was used to capture calcium signaling (Figure 6). Briefly, side chambers of the chips were loaded with Rhod-3 AM dye and washed, and time-lapse images were captured during the application of PUFFS. Within any experiment, some cells displayed spontaneous Ca2+ activity, prior to PUFFS application, likely reflecting other cellular processes. For every recording session, PUFFS were applied 3 times and separated by 3 rest periods (3 min long), and regional calcium signaling responses were plotted as spectrograms, as described in the Materials and Methods. For cocultured chips, spectrograms show high osteocyte activity (yellow pixels) during the 3 instances of PUFFS as cells try to match the stimulation frequency (∼0.3 Hz). Also, the activity during PUFFS decreases from regions proximal to PUFFS to those distal to PUFFS within OCY in Ch#2, while the lowest activity is recorded in Ch#1 (the location of the MC3T3 culture). For OB-only chips, no cell activity was detected, while for OCY-only chips, we see a similar trend of decreasing cell activity from proximal-to-distal regions with respect to PUFFS. Results show that cells in Ch#2 are more active than cells in Ch#1. It is worth noting that the recorded signals could be a combination of 3D OCY454s and any MC3T3s that migrated into migrated Ch#2 (see Figures 2 and 5), which could not feasibly be distinguished in this experiment. Apart from stimulus-evoked Ca signal activity during PUFFS, spontaneous calcium signaling activity was observed in some cells. Based on our previous work, cell-laden collagen in the central chamber of the chip (Ch#2) will experience stress that varies from 56.17 kPa (near PUFFS, Ch#3) to 11.23 kPa (away from PUFFS, Ch#1).^69^ This could explain the detection of greater calcium signaling in the osteocytes located in the central chamber, closer to the application of PUFFS signaling in Ch#3. This also explains a lack of calcium signaling activity for MC3T3s, which are seeded in Ch#1 away from PUFFS. Although we do see some activity in the OCY-only chips, it is much less than that in the OB-OCY chips, suggesting that coculture conditions are necessary to sense PUFFS in this model. This result is in line with other studies that show enhanced calcium signaling and responsiveness of cocultured OB-OCY to mechanical stimuli as compared to monocultures.^77^

**Figure 6:**
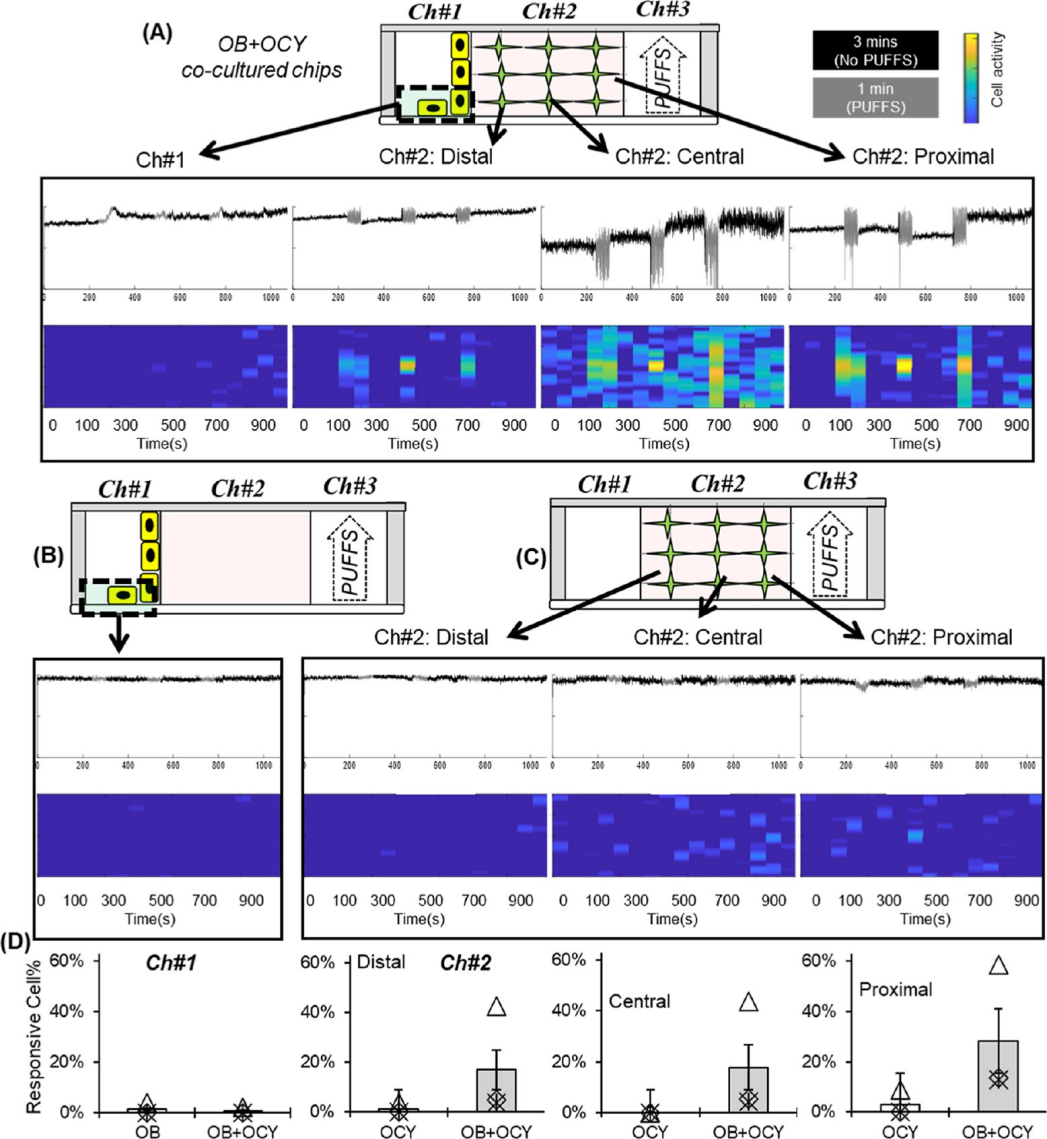
(A) Schematic and representative normalized calcium signaling profiles showing cell activity in proximal, central, and distal subregions with respect to PUFFS in a cocultured (OB + OCY) chip. (B) Representative schematic and signaling in OB-only chips. (C) Representative schematic and calcium signaling in subregions of OCY-only chips. (D) Number of responsive cells, with oscillating signals, in mono-Vs cocultured chips on Day 31 in Ch#1 and subregions of Ch#2.

### Characterization of Sequentially Cocultured Chips with Model Osteoclasts

On Day 31, cells from Ch#1 (possibly a mix of seeded MC3T3-E1 and OCY454s migrated from Ch#2) were removed using a dilute trypsin solution, and preosteoclasts RAW264.7 cells (2 × 10^5^ cells/ml) were pipetted into Ch#1 and cultured for 3 days using osteoclastic media under daily PUFFS and under static control conditions. Cell morphology (f-actin, nuclei) and osteoclast differentiation (TRAP+) were characterized on Day 34. Like mono- and coculture conditions, composite bright-field and fluorescence images show DMP1-GFP expression within OCY454-laden collagen gels in Ch#2 for both PUFFS and static control conditions (Figure 7B). Cell viability within chips, assessed using a live–dead staining assay, shows high cell viability for both PUFFS (82% ± 15%) and static controls (76% ± 26%) on Day 34, with no significant differences between groups. Cell morphology, based on f-actin and nuclei staining, shows RAW264.7s with multiple nuclei, indicating osteoclast differentiation within Ch#1 of chips. Positive staining for tartrate-resistant acid phosphatase (TRAP) also confirmed osteoclast-specific activity within the chip. Representative high-resolution images from Ch#1 show the presence of multiple nuclei, indicating osteoclast differentiation and maturity. As compared to PUFFS chips, TRAP-positive cells under static culture conditions exhibit an increase in the cell area, although the difference is not significant. Gene expression for osteoclastic genes associated with resorption-dependent adhesion (TRAP and ITGB3) and differentiation (NFATc1 and RANK) was characterized using RT-qPCR. There was no significant difference in groups for either TRAP (0.66-fold, *p*_(ΔCT)_ = 0.833) or ITGB3 (2.1-fold, *p*_(ΔCT)_ = 0.4685). Changes in NFATc1 (6.4-fold, *p*_(ΔCT)_ = 0.0455) and RANK (41.9-fold, *p*_(ΔCT)_ = 0.0351) point to osteoclast differentiation within Ch#1 of the chips. We suspect that this finding for NFATc1 expression in osteoclastic cells could reflect a long-term preconditioning effect of mechanical loading on the secretory activity of the osteocytes that were previously in culture alongside osteoblasts. Classic studies show that physiological levels of the fluid shear stimulus in vitro shift the RANKL/OPG ratio to restrain osteoclast formation, while static conditions promote osteoclast formation. It bears noting, however, that these studies are generally of short duration (1–5 days) of stimulation in monolayer culture. In contrast, in vivo studies, generally of longer duration, paint a much more complex picture where loading stimuli can simultaneously promote and suppress osteoclastogenesis at adjacent locations depending on regionally variable strain conditions. High-strain conditions favor pro-osteoclastogenic signaling (via RANKL, IL-6 family, prostaglandins, etc.), vice versa for low-strain environments.

**Figure 7:**
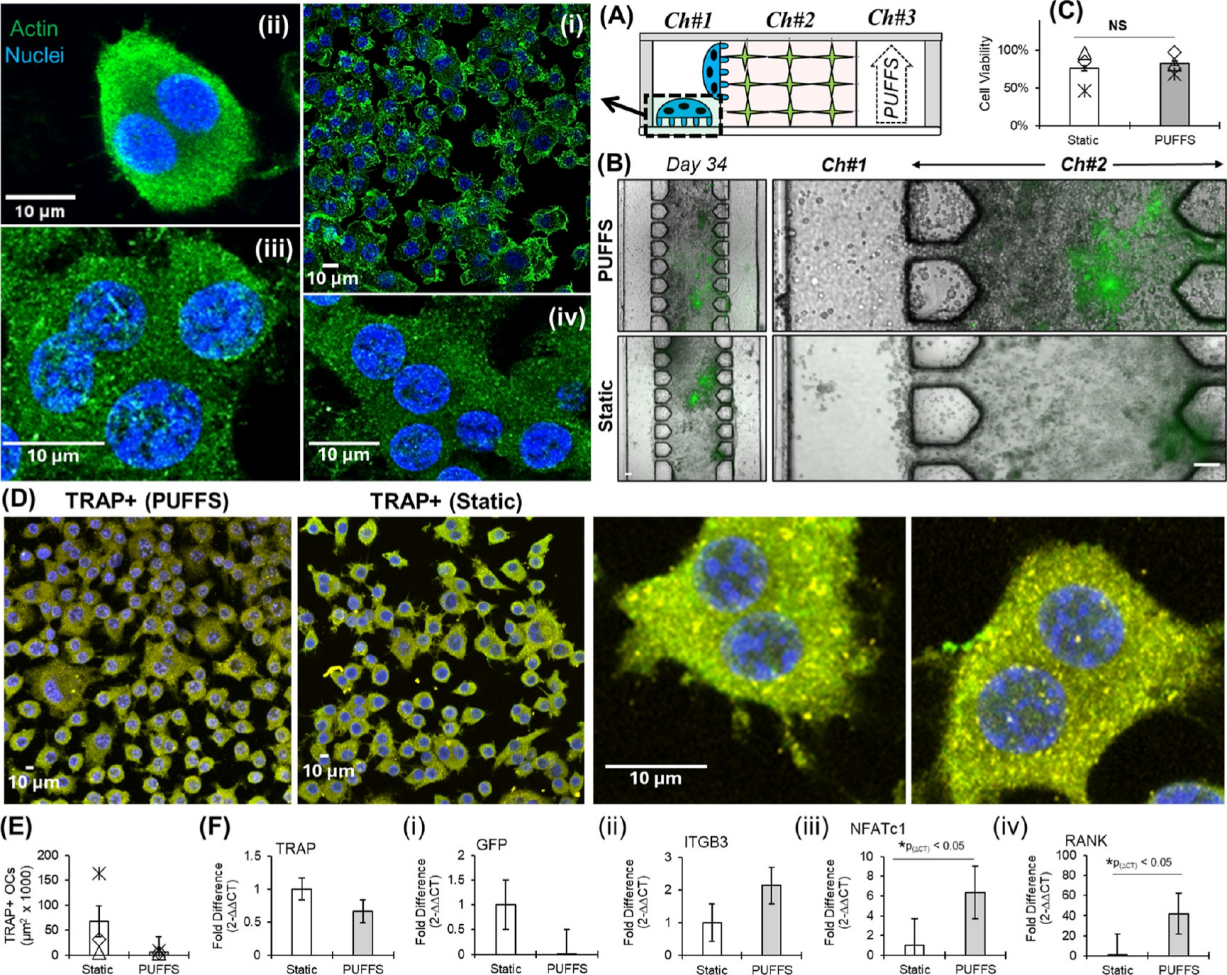
(A) Schematic showing the cross-section of the chip on Day 34 with OCs (RAW264.7) in Ch#1. (i–iv) Morphology of OCs in Ch#1 at different resolutions (f-actin = green; nuclei = blue). Scale bar = 10 μm. (B) Composite bright-field and fluorescence images with 3D OCY454 networks in Ch#2 under PUFFS and static control conditions. Scale bar = 100 μm. (C) Cell viability within chips subjected to PUFFS and static controls on day 34. (D) Representative images show the TRAP signal (yellow) for PUFFS and static control after 3 days of osteoclastic differentiation in Ch#1. Scale bar = 100 μm. (E) Plot showing area of TRAP + cells for PUFFS and static conditions on Day 34 in Ch#1. (F) (i–v) RT-qPCR plots of relevant genes. Data mean based on ΔCT values between PUFFS and static control from *n* = 3–6 replicates. Significance determined by one-way ANOVA.

## Discussion

The holy grail of replicating skeletal remodeling on a chip is to combine dynamic mechanical stimulation with a culture of mechanosensing osteocytes, bone-forming osteoblasts, and bone-resorbing osteoclasts into a single integrated model. In earlier work, we established a simple microfluidic monoculture model of osteocyte mechanotransduction, wherein we used live-cell fluorescence microscopy to evaluate Ca2+ signal propagation across self-assembled 3D networks of collagen-entombed murine osteocytes (MLO-Y4) in response to physiologically relevant bouts of PUFFS stimulation for a period of up to 2 weeks.^55^ Unlike static models or simple monolayer fluid shear models, our PUFFS better replicate in vivo mechanical stimulation by providing both interstitial fluid flow and gradients of matrix deformation, which activate distinct mechanotransducive pathways that osteocytes integrate to coordinate the mechanoadaptive activity of OB and OC.

The three-chambered chip design of our initial model was conceived to enable addition of one or more cell types to the system. Building on this initial design, the current study presents a more advanced multicellular unit (BMU)-on-chip model that incorporates osteoblastic (MC3T3-E1.4, OB) and osteoclastic (RAW264.7, OC) cells alongside 3D networks of collagen-matrix-entombed (OCy, OCY454) and enables investigation of the mechanisms by which mechanical forces are transduced into bone metabolic responses that in turn determine the bone structure and material properties. The current work validates this approach by establishing key parameters for co- and staged sequential coculture models, in situ differentiation of preosteoblasts and preosteoclasts, and sustained expression of key functional markers typical of each cell line when subjected to daily bouts of PUFFS for periods of up to one month. Bone remodeling is a tightly coupled process where osteoclasts and osteoblasts operate in a specific sequence at any single spot of bone turnover rather than simultaneously. Guided by our previous work,^69^ OCY-only chips were allowed to generate robust connections in collagen for 9 days before adding any other cells to the chip. To mimic sequential coupling observed during bone remodeling, osteoblasts (MC3T3s) were added in Ch#1 on Day 9, cultured under osteogenic conditions, and subjected to PUFFS until Day 31 to simulate the bone formation phase of bone remodeling. On Day 31, OBs were trypsinized and removed, and OCs were added to simulate the resorption phase of remodeling. The late addition of the OCs was, in part, motivated by practical considerations, as the RAW264.7 cells proliferate rapidly (doubling time of 12–15h), and would quickly overpopulate the system. Since the focus of this work is to demonstrate the capability of adding and removing relevant cells to/from defined chambers and at user-defined times/sequence, we did not test a specific hypothesis related to the influence of preconditioning of the OCY chips with OBs before introducing OCs, although this can be tested in future experiments. This provides a high degree of modularity over experimental design. The chip also provides spatiotemporal control over the cellular microenvironment such as cell seeding/removal from Ch#1, 3D culture of OCY in Ch#2, and application of PUFFS using Ch#3.

Beyond the addition of osteoblastic and osteoclastic cells, several key refinements were made in establishing our advanced BMU-on-Chip model. Our earlier model used the MLO-Y4 osteocyte-like cell line, which readily adopts the 3D lacuna-canalicular morphology similar to osteocytes entombed in bone and is responsive to mechanical stimuli by transmitting intracellular Ca2+ via C*x*43 gap junctions.^56–59^ However, the MLO-Y4 cell line is an imperfect osteocyte model in that it represents a very early stage in the continuum of osteoblast-to-osteocyte maturation and, at baseline, expresses very low or undetectable levels of key proteins that label the vast majority of osteocytes in vivo (DMP1, FGF-23). Furthermore, unlike osteocytes in vivo or primary bone-derived osteocytes, MLO-Y4 is an immortalized cell line, and its high level of proliferative activity confounds long-term experiments that rely on visualization signal propagation between individual cells.

In the current work, we selected the conditionally immortalized OCY454 cell line, which sustains proliferation at permissive temperatures (33 °C) but enters an arrested quiescent state at 37 °C, allowing longitudinal live-cell and time-lapse experiments. Additionally, the OCY454 line expresses a maturation-dependent GFP reporter driven by the DMP1-promoter, which we have now shown can be accelerated by mechanical stimulation. Likewise, in this model system, long-term monoculture of OCY454 or coculture with MC3T3-E1.4 osteoblasts and asynchronous sequential coculture with rank-L-induced osteoclasts under prolonged mechanical stimulation preserves expression of key markers of each lineage (Figures^3^, S3, and^7^).

Our earlier study used a collagen concentration of 2.5 mg/mL, which allowed rapid establishment of lacuna-canalicular networks connecting adjacent cells but routinely delaminated from the microfluidic device in experiments longer than 14 days and also permitted excessively high levels of OB migration into the collagen matrix (Figure S7). To address this, in the current study, we increased the concentration of collagen used to suspend the osteocytes in the initial steps of preparing the BMU-on-chip devices, without compromising self-assembly of 3D networks, offered better preservation of the Ocy-Ob interface, and did not delaminate in long-term studies or following enzymatic cell removal.

While this work has focused on validating a model that allows investigation of the interaction of the three primary bone cell types, we envision future iterations of this platform would incorporate additional cell lineages that represent the vascular, nervous, hematopoietic, and marrow stromal lineages to more faithfully recapitulate the bone microenvironment. Furthermore, PUFFS can be dynamically modulated across comparison of a range of physiologically relevant parameters such as load-magnitude, loading frequency and waveform, duration, and periodicity of loading/unloading bouts. These parameters can be tuned to replicate a mechanotransducive stimulus ranging from high-load (resistance training) and high-intensity exercise to modeling a sedentary lifestyle or even modeling the cellular biology underlying disuse osteopenia, and could feasibly be combined with pharmacological interventions.

As with every reductionist model, the BMU-chip also has limitations. The ideal medium composition for the coculture of cells within these chips remains a key challenge, as diffusion of the medium through collagen is expected to occur and might be unfavorable to certain cell types. For instance, a proosteoclastic medium could inhibit the normal functioning of OCY454; this needs to be characterized further. While a nonmineralized and transparent collagen matrix allows the use of time-lapse microscopy to capture the dynamic calcium signaling response during PUFFS, future studies should consider the role of mineralization. Culture conditions with MC3T3 could be optimized to enable mineral deposition before adding preosteoclasts or collagen in the central chamber, which could be artificially mineralized using simulated body fluid, followed by seeding and culture of OCs to study osteoclastogenesis. This model utilizes a diluted trypsin solution to remove cells from Ch#1, and the effects of these washing steps on cells within various chambers of the chips could be systematically characterized. Similarly, cell concentrations during both seeding (OB, OC) and encapsulation (OCY) can also be optimized; for instance, in the current model, we observe that the OCY454s aggregate on top of each other within 3D collagen by Day 31 in some regions of Ch#2, something never seen in native tissues. Lastly, the use of human-derived cells instead of model cells could significantly advance our understanding of osteocyte mechanotransduction and its role in bone remodeling.

In summary, our current BMU-on-chip balances tradeoffs between experimental flexibility and throughput of traditional in vitro models, against the physiological relevance and experimental challenges of animal models. By incorporating the three predominant bone cells (OCy, OB, and OC) and defined cyclic loading conditions, we have established a systematic method to study complex cellular crosstalk related to bone remodeling, specifically in response to mechanical stimuli. Unlike 2D cultures, this chip encapsulates OCY454s within a collagen matrix, which over the culture duration self-assembles into 3D interconnected networks. An addition would be to include a comparison with other microfluidic platforms that enable the study of mechanical forces on cells and the distinct advantages of the system presented here. Table S3 in the Supporting Information highlights key differences between this work and the state of the art. A key feature of this work is its modularity. Both cell seeding and application of PUFFS can be potentially applied from either side chamber. The microfluidic chip provides precise spatiotemporal control over the microenvironment, which can allow for the study of specific cellular interactions and signaling pathways. In the short term, this chip can be used to study isolated phases of the remodeling cycle, while in the long term, we envision the full recapitulation of the bone remodeling cycle with potential use in drug screening. By elucidating how osteocytes coordinate osteoblast and osteoclast activities in response to mechanical loading, such models, when combined with human-derived cells, can identify new mechanisms of bone diseases involving aberrant mechanotransduction and reduce our reliance on animal models.

## Materials and Methods

### Design and Fabrication of Multichambered Chips Using 3D Printed Master Molds

For 3D printing master molds, glass slides (25 × 75 × 1 mm, Fisher brand) precleaned with a piranha solution (H_2_SO_4_ and H_2_O_2_; 7:3, stirred for 30 min at 125 rev/min) were neutralized and dried in a vacuum oven at 65 °C and then further modified in a 9:1 solution of 3-(trimethoxysilyl)­propyl methacrylate (TMSPMA; Sigma-Aldrich) and toluene (Sigma-Aldrich). A carbide-metal etching pen was used to slice the dried modified glass slide into 4 pieces, and double-sided tape was used to adhere each glass piece onto an aluminum print block to be screwed into the printer stage head. The prepolymer solution was formulated with 40 mL of poly­(ethylene glycol) diacrylate (PEGDA, average Mn 250) and 0.25% of the photoinitiation agent (Irgacure 819, Sigma-Aldrich), 0.01% of TEMPO (Sigma-Aldrich), and 0.5% of 2-isopropylthioxanthone (ITX, Tokyo Chemical Industry). Then, the solution was vortexed for 10 min and stored until further use in the dark (wrapped with aluminum foil to prevent exposure to light). A custom-built Projection Stereolithography (PSLA) setup was used to print the master molds—a replica of the intended microfluidic chip design. Based on the mold design, CAD files were generated using Fusion 360. An optical printer built using Digital Light Projection (DLP, development kit 1080p 9500 UV, Texas Instruments, USA) was used to print master molds onto treated glass slides. The total height of the printed mold, set to 250 μm, was confirmed with a digital caliper (Mitutoya). Then, replica casting was used to develop multiple master molds using polydimethylsiloxane (PDMS) to minimize batch variation. Briefly, the PDMS elastomer was mixed for 10 min at 10:1 with the curing agent (Sylgard 184, Dow Corning Silicone Elastomer), degassed in a desiccator, poured evenly on at least 5 PEGDA master molds in a 100 × 15 untreated Petri dish, and allowed to polymerize at 70 °C overnight. The PDMS sheet was peeled off, and six 2 mm holes were generated to create the inlet/outlet ports using a biopsy punch. Then, PDMS sheets were incubated in 100% ethanol and plasma-bonded to 22 mm × 22 mm glass coverslips (PCS-1.5–2222, Mattek) pretreated with 30% hydrochloric acid wash (overnight incubation). The bonded chips were placed on hot plates at 150 °C for an hour to ensure robust bonding. Chips were brought to room temperature before incubating them in 100% ethanol, followed by overnight sterilization in a BSL-2 cell culture hood under UV light.

### Mono- and Coculture in Chips

Before cell incorporation, the chips were surface-modified as follows: (i) 2 mg/mL polydopamine (PD) (Sigma-Aldrich, no. H8502) solution was pipetted into Ch#2, incubated at room temperature for 24 h, and then washed with PBS (3×). (ii) Chips were incubated in a 0.01% poly l-lysine solution (PL) (Sigma-Aldrich, #P4707) for 15 min at room temperature and washed with PBS (3×) followed by another coating of 0.15 mg/mL rat tail type 1 collagen for an hour at room temperature. (iii) The central chamber (Ch#2) was washed with PBS (3×), dried, and sterilized under UV radiation for 45 min before incorporating the osteocyte cell solution. Subconfluent OCY454 osteocytic cells^56^ were cultured in alpha-MEM (#12571063, Gibco) containing l-glutamine, 1% Anti-Anti (Life Technologies, #15240), and 10% fetal bovine serum (S11150, GeminiBio) on surface-treated flasks (Thermofisher, 130190). Cells were cultivated at 33 °C under a humidified atmosphere of 5% CO_2_ to maintain proliferation. Upon reaching 90% confluency, cells were harvested (TyrpLE, Life Technologies, #12605-010) and resuspended in media and mixed with a collagen solution. To prepare the 4 mg/mL collagen solution, 1333 μL of bovine type 1 collagen (no. 5225 bovine, Advanced BioMatrix, 6 mg/mL) was mixed with 314 μL of 10× HBSS (ThermoFisher, no. 14065056), 236 μL of a neutralizing agent (Advanced BioMatrix), and 120 μL of the resuspension medium with 7.2 × 10^6^ cells/mL. A 10 μL aliquot of the collagen solution homogeneously mixed with ∼1.3 × 10^4^ of the OCYs was pipetted into Ch#2 for each chip and incubated at 33 °C for 30 min to gel the collagen matrix, while Ch#1 and Ch#3 were filled with media and replenished daily. Ch#1 was precoated with a 5 μg/mL fibronectin solution (Thermofisher, 33010018) for 2 days after OCY454 encapsulation. Chips were cultured at 33 °C for 3 days, after which the chips were maintained at 37 °C for the duration of the experiment. MC3T3-E1 preosteoblasts (OB) (ATCC), maintained in an ascorbate-free alpha-MEM medium (no. A1049001, Gibco) supplemented with l-glutamine, 1% penicillin/streptomycin, and 10% fetal bovine serum, were cultured in surface-treated flasks and incubated at 37 °C under a humidified atmosphere of 5% CO_2_. Nine days after OCY454 encapsulation, near-confluent (75%–90%) MC3T3-E1.4 cells were trypsinized (0.05% Trypsin + 0.53 mM, Life Technologies, #25300062), resuspended to 1 × 10^6^ cells/mL, and seeded in Ch#1 (3 × 10^4^ cells per chip). The following day, osteogenic induction media with 100 μM l-ascorbic acid-2-phosphate (Sigma-Aldrich), 5 mM β-glycerophosphate (Sigma-Aldrich), and 10 nM dexamethasone (Sigma-Aldrich) were exchanged in Ch#1 daily for 21 days. From day 11, PUFFS were applied for 15 min daily in Ch#3 until the target end point. For sequential coculture experiments, cells in Ch#1 were removed using a 0.05% trypsin-EDTA solution on day 31, and the RAW264.7 preosteoclast cell solution (10 μL) was pipetted. These cells were maintained in DMEM (Thermofisher, #11995065) containing 10% fetal bovine serum, 1% penicillin/streptomycin, and 1% Glutamax (Thermofisher, #35050061) and were resuspended to a density of 2.4 × 10^5^ cells/mL. Then, an osteoclastic medium with 20 ng/mL RANK-L (R&D, #462-TEC) and 50 μg/mL vitronectin (Thermofisher, A14700) was introduced in Ch#1, and PUFFS (15 min per day) was used in Ch#3. All PUFFS experiments were performed at room temperature in a sterilized biosafety cabinet level 2 (BSL-2). After PUFFS, the medium was replenished, and chips were cultured under standard conditions (37 °C and 5% CO_2_). Static-control chips were not subject to PUFFS.

### Recording of Calcium Signaling and Analysis

Imaging of PUFFS-evoked calcium signaling was performed on day 31 for both monocultures (OB-only, OCY-only) and cocultured (OB + OCY) chips. For reproducible calcium signal measurements, device stabilizers were designed and developed via CAD-fusion360 and 3D printed using poly­(lactic acid) (FDM, Bambu Lab P1P equipped with a smooth PEI bed plate set at 65 °C–250 °C and 0.4 mm temperature nozzle). For each individual chips for each treatment group, side chambers (Ch#1, Ch#3) in Petri dishes (35 mm × 10 mm) with adapters were loaded with a calcium dye solution (500 μL, media +0.02% PowerLoad +0.01% Rhod-3 AM, #R10145, Thermofisher), then covered with aluminum foil, and incubated at 37 °C for 45 min. Sterile plastic connectors, created by slicing along the upper marked sections of 1000 μL micropipet tips, were connected at the inlet/outlet ports of Ch#3.

For each session, changes in calcium intensity from cells were recorded using fluorescent microscopy (Leica DMi8 Inverted Confocal, 10× objective) at a single plane within the region of interest (ROI). Here, the ROI was chosen to be a plane at ∼100 μm from the bottom glass coverslip (approximate center plane of ∼250 μm thick OCYs in collagen in Ch#2). Before testing, the pump was connected to the chips’ inlet and outlets and left undisturbed for 18 min. For each calcium signaling experiment, baseline data was captured for the first 40 s before PUFFS loading (3 min for 60 s at 0.33 Hz), totaling PUFFS application 3 times until the end of recording. Time-lapse images were captured during the application of PUFFS. Recordings were exported as .Lif/.LOF files; pre- and postprocess analyses were done with ImageJ software, Excel, and MATLAB. To acquire calcium intensity activity, experiment recordings were imported as LOF/LIF files into ImageJ for automation of fluorescence intensity quantification for all time frame stacks (Figure S4). Here, the *z*-projected mask outline was used to capture the displacement of individual cells based on the change in fluorescence throughout all image stacks, and an autothreshold was then applied to identify and add cells to the ROI manager. The nonfluorescence regions with no cells were used as background control. The resulting data (mean, area, integrated density) after selecting the multimeasure option to analyze intensities within the outlined regions for all image stacks of single sample was exported as a .CSV file into Excel to calculate the corrected total cell fluorescence (CTCF) = Integrated Density (Area of Selected Cell × Mean Fluorescence of Background readings), where the change in calcium intensity is fluorescence over the initial baseline fluorescence (F/F0). A similar process was used in our previous work.^55^ Individual cell signaling data were combined to generate regional signaling responses, and MATLAB (DistSigPlot_FN.m) was used to plot the results as spectrograms.

### Gene Expression Using RT-qPCR

RNA isolation was done with two chips for identical conditions to obtain a robust RNA yield, and all of the following procedures were done on ice. Ch#1 of OB-only chips and OB + OCY chips were washed with PBS, and then cells were trypsinized and transferred to a single 2 mL vial. The culture medium was added at a matching volume to the vial to deactivate the trypsin. Suspended cells were centrifuged at 5000*g* for 5 min. The supernatant medium was aspirated, leaving the pellet undisturbed. To isolate RNA, the isolated cells were homogenized in 300 μL of lysis buffer (12183555, Invitrogen) by vortexing for 1 min. Samples were left to incubate over ice for 15 min to permit complete dissociation. 70% ethanol was added at equal volume and then vortexed for less than 10 s to mix well. The RNA was extracted by the recommended protocol with adjustments to the starting volume (∼600 μL), including Wash Buffer I (350 μL) and Wash Buffer II (200 μL first cycle, 100 μL second cycle), and further purified using RNA purification kit columns (#12183555, Invitrogen). For sequentially cocultured chips, RAW264.7 (OCs) were isolated from Ch#1 using a similar method. For the OCY-only chips, the medium was gently removed and washed with 1× PBS, Ch#1 and Ch#3 were incubated in TRIzol for at least 5–10 min to solubilize the OCY-laden collagen, and the remaining steps for extraction were followed as described by the kit. The RNA recovery volume was 30 μL. RNA purity and quantity were assessed by NanoDrop spectrophotometry (Thermofisher). The isolated RNA (35 ng/sample) was reverse-transcribed to cDNA (Quantitect Reverse Transcription Kit, Qiagen). Synthesized cDNA frozen at −80 °C was thawed and then amplified with (Quantitect SybrGreen PCR Kit, Qiagen) and oligonucleotide primers (Tables S1 and S2; Azenta Life Sciences), using an Applied Biosystems SimpliAmp Thermocycler and QuantStudio3 Real-Time PCR instrument, respectively. For qPCR analysis, any value with a CT higher than 45 was considered undetectable and assigned a 0 value. Following qPCR, data was normalized using the geometrical mean of two housekeeping genes­(HKG) (beta Actin and GAPDH) expression and reported as RNA relative to HKG (Delta CT). Data was analyzed using a two-way ANOVA with Kruskal–Wallis test and multiple comparisons. Data was analyzed using Prism 10.

### Cell Viability, Morphology, and Immunostaining

For cell viability, chips were incubated with 0.2% calcein AM and 0.1% ethidium homodimer-1 solutions, washed with PBS, and imaged using a Leica DMI6000 Inverted microscope. At specific time points, cells in chips were fixed in situ using 4% formaldehyde in PBS for 15 min at room temperature, washed with PBS by perfusing PBS in Ch#1 and Ch#3 three times, followed by permeabilization using 0.2% Tween for 10 min, washed 3 more times, blocked with 1% BSA for 1 h at room temperature, and then soaked in PBS for 15 min twice. Chips were incubated with primary antibodies at 4 °C overnight and then incubated with a secondary antibody solution prior to fluorescence microscopy imaging. Morphology was assessed using f-actin (Alexa 488 phalloidin, Thermofisher) and nucleus stain (1 μg/mL DAPI, diamidino-2-phenylindole, Thermofisher) and then imaged using an upright Leica DM6 B fluorescence microscope equipped with a THUNDER tissue imager. For immunostaining, the following primary and secondary antibody solutions were used in target microchambers. Primary antibodies were diluted in 0.2% BSA, 0.1% Tween, 0.3% Triton-X 100 (BTT), 1:50 dilution of Mouse Monoclonal Runx2 antibody (#H00000860-M01, Thermofisher) in Ch#1, 1:100 dilution of Rabbit Polyclonal TRAP (#PA5116970, Thermofisher) in Ch#1, and 1:100 dilution of Chicken Polyclonal GFP (#A10262, Thermofisher) in Ch#3. Secondary antibodies were diluted in BTT at 1:400 dilution: Alexa Fluor Plus 647 Goat anti-Mouse IgG secondary antibody (no. A32728, Thermofisher), Alexa Fluor 594 Goat anti-Rabbit IgG (H + L) Highly Cross-Adsorbed secondary Antibody (no. A32740, Thermofisher), and Alexa Fluor Plus 405 Goat Anti-Chicken IgY Cross-Adsorbed secondary (#A48260, Thermofisher). ImageJ was used to process images.

### Ethical Statement

All experiments were done in compliance with the approval of the Syracuse University Institutional Biological Committee. OCY454 cells were isolated from double-transgenic mice, as previously described (PMID: 25953900), and all animal experimental procedures were approved by the Institutional Animal Care and Use Committee (IACUC) of Massachusetts General Hospital and Boston University.

### Statistical Analysis

One-way and two-way ANOVA/Tukey tests were conducted using SAS software to identify significant differences. PCR plots were statistically analyzed with GraphPad Prism (10.4). *p < 0.05, **p < 0.01, and ***p < 0.001 were considered statistically significant.

## Supplementary Material





## Notes

Additional experimental details, methods, including photographs of experimental setup, analysis workflow, tables listing primer sequences, and a caption for the video file (PDF)
Video S1 (MP4)
