# Supplementary material for: Bone Multicellular Unit on a Chip (BMU-Chip) Subjected to Cyclic Mechanical Loading
Source: ACS Biomater Sci Eng. 2026 Mar 11;12(4):2301–14. doi: 10.1021/acsbiomaterials.5c01798 (PMC13080768; doi:10.1021/acsbiomaterials.5c01798)
Supplement: Supplementary file 1 [file ab5c01798_si_001.pdf]

Supplementary Materials For

Bone Multicellular Unit on a chip (BMU-chip) subjected to cyclic mechanical loading

Anna-Blessing Merife[1], Michael P. Seitz[1], Angelika Polshikova[1], Ujjwal Aryal[1], Zachary J. Geffert[1], Era Jain, Jason Horton[3], Paola Divieti Pajevic[2], Pranav Soman[1#]

[1]Department of Chemical and Biomedical Engineering, Syracuse University,

[2] Department of Translational Dental Medicine, Goldman School of Dental Medicine, Boston University.

[3] Department of Neuroscience and Physiology, Alan and Marlene Norton College of Medicine, SUNY Upstate Medical University, Syracuse, New York 13210, United States.

[#] corresponding author: psoman@syr.edu

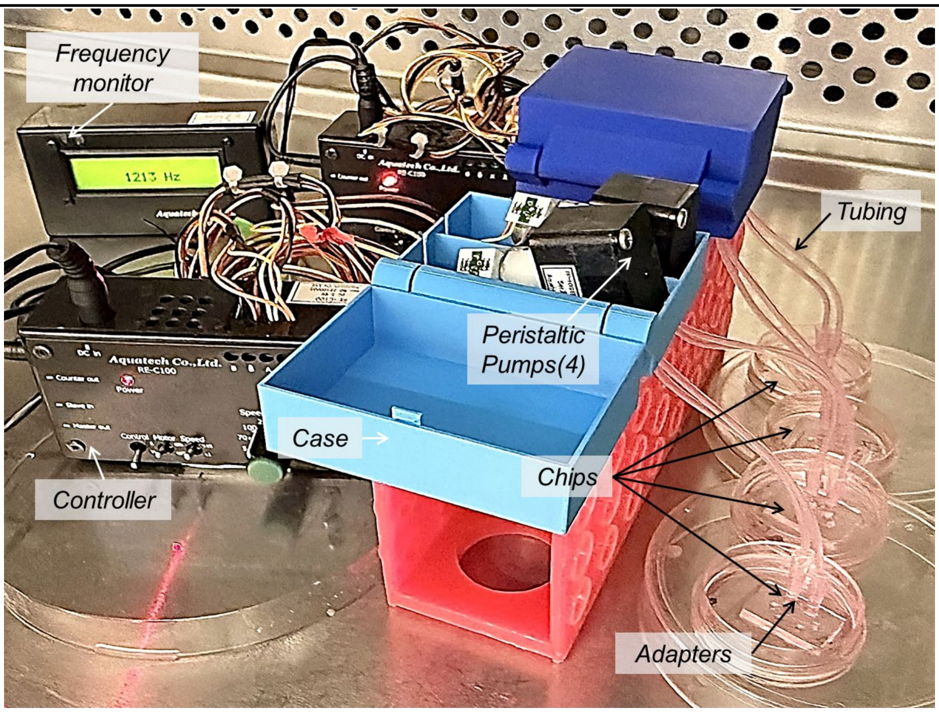

Figure S1 Picture of perfusion setup showing 2 peristaltic pumps held within 3D printed case. This setup is used to apply PUFFS (0.33Hz) to chamber # 3 of 4 independent PDMS chips using adapters, tubing, frequency monitor and controller.

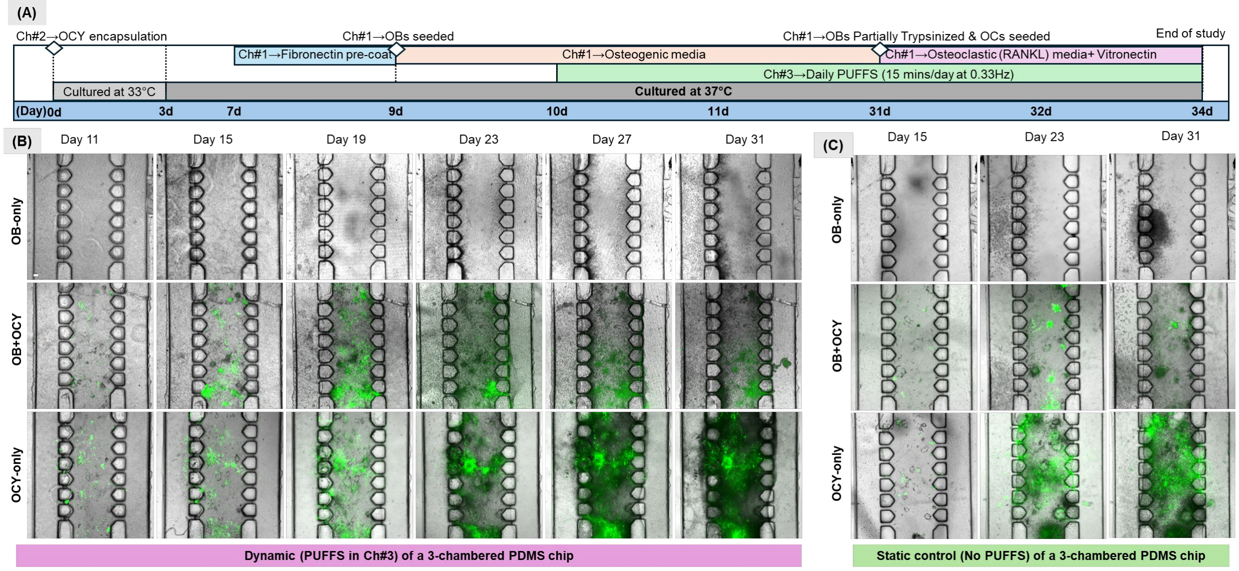

Figure S2 (A) Study workflow, and representative composite images (brightfield + fluorescent) images for selected time-points showing OCY454 expressing DMP-1 (GFP) in chamber 2 of chips subjected to PUFFS (B) and chips used as static controls (C).

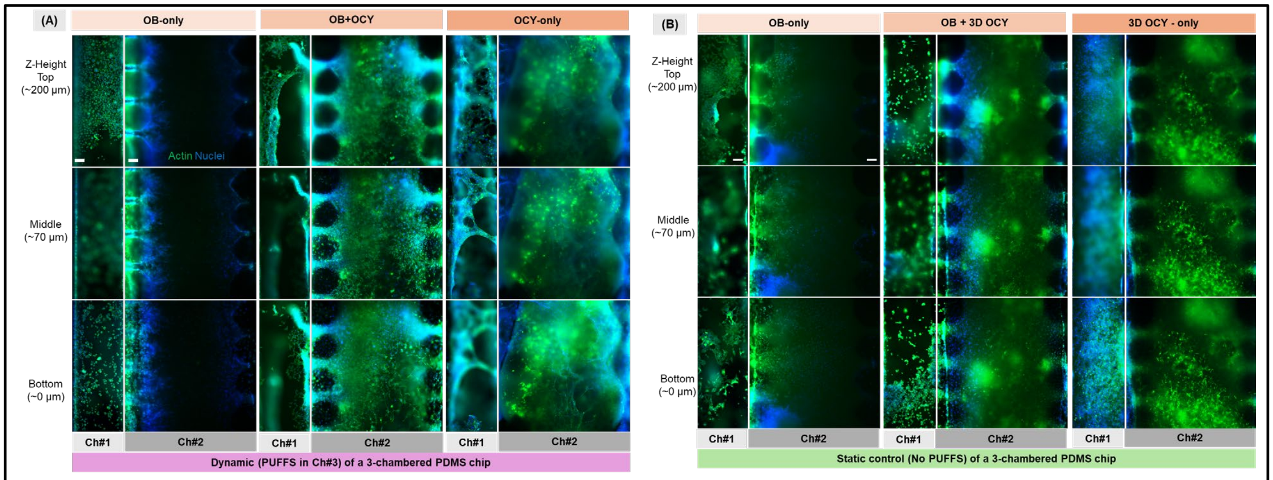

Figure S3 Representative images of OCY morphology captured at three z-planes from the bottom glass coverslip (0 µm, ~70 µm, ~200 µm) for chips subjected to PUFFS **(A)** and static controls **(B)** (Green = f-actin; Blue = nuclei; Scale bar = 100 µm)

Gene	Symbol	OB v. OB+OCY Fold Change ($2^{-\Delta\Delta CT}$)	OB v. OCY Fold Change ($2^{-\Delta\Delta CT}$)	Relevance	Primer sequence
Alkaline Phosphatase	*ALP*	84.4	1355.7	Bone growth development and regulates mineralization	F:5'-CCAACTCTTTTGTGCCAGAGA-3'<br>R: 5'-GGCTACATTGGTGTTGAGCTTTT-3'
Osteopontin	*OPN*	4.8	0.0	Bone metabolism and homeostasis; mineralization inhibitor	F: 5'-AGCAAGAAACTCTTCCAAGCAA-3'<br>R: 5'-GTGAGATTCGTCAGATTCATCCG-3'
Runt-Transcript factor 2	*RUNX2*	77.1	5.9	Osteoblast differentiation marker; shapes skeletal structure	F: 5'-ATGCTTCATTCGCCTCACAAA-3'<br>R: 5'-GCACTCACTGACTCGGTTGG-3'
Osterix	*Sp7**	4.3	1508.6	Osteoblast differentiation marker	F: 5'-ATGGCGTCCTCTCTGCTTG-3'<br>R: 5'-TGAAAGGTCAGCGTATGGCTT-3'
Collagen type I alpha 1	*COL1A1*	3.7	63.4	Promotes structural integrity	F: 5'-GCTCCTCTTAGGGGCCACT-3'<br>R: 5'-CCACGTCTCACCATTGGGG-3'
Osteoprotegerin	*OPG*	1.1	0.1	Involved in remodeling; inhibits resorptive activity	F: 5'-ACCCAGAAACTGGTCATCAGC-3'<br>R: 5'-CTGCAATACACACACTCATCACT-3'
Fibroblast growth factor 23	*FGF-23*	1.3	0.0	Regulates phosphate levels	F: 5'-ATGCTAGGGACCTGCCTTAGA-3'<br>R: 5'-AGCCAAGCAATGGGGAAGTG-3'
Dentin Matrix Protein 1	*DMP1*	30222.1	10722.2	Regulates mineralization and osteoblast activity	F: 5'-AAAGACCACGACAGTGAGGAT-3'<br>R: 5'-CATCATCGAACTCAGAACCGTC-3'
Phosphate-regulating endopeptidase homolog X-linked	*PHEX*	0.0	12.6	Regulates phosphate levels	F: 5'-GAAAGGGGACCAACCGAGG-3'<br>R: 5'-AACTTAGGAGACCTTGACTCACT-3'
Osteocalcin	*BGLAP*	1.1	101.8	Involved in remodeling and energy metabolism	F: 5'-CTGACCTCACAGATCCCAAGC-3'<br>R: 5'-TGGTCTGATAGCTCGTCACAAG-3'
β-Catenin	*CTNNB1*	7.8	0.1	Involved in cell adhesion, growth and development	F: 5'-ATGGAGCCGGACAGAAAAGC-3'<br>R: 5'-CTTGCCACTCAGGGAAGGA-3'
GFP				Identify Ocy454 cells	F: 5'-ACTACAACAGCCACAACGTCTATATCA-3'<br>R: 5'-GGCGGATCTTGAAGTTCACC-3
		GAPDH		HSK	F: 5'-AGGTCGGTGTGAACGGATTTG-3'<br>R: 5'-TGTAGACCATGTAGTTGAGGTCA-3'
		HPRT1		HSK	F: 5'-TCAGTCAACGGGGGACATAAA-3'<br>R: 5'-GGGGCTGTACTGCTTAACCAG-3'
		Actin-β		HSK	F: 5'-GGCTGTATTCCCCTCCATCG-3'<br>R: 5'-CCAGTTGGTAACAATGCCATGT-3'

Housekeeping Genes (HSK) = Average CT value GAPDH, HPRT1, BetaActin

Table S1 Oligonucleotide primers and relative fold-change of gene expression by RT-qPCR for monocultures and cocultured chips.

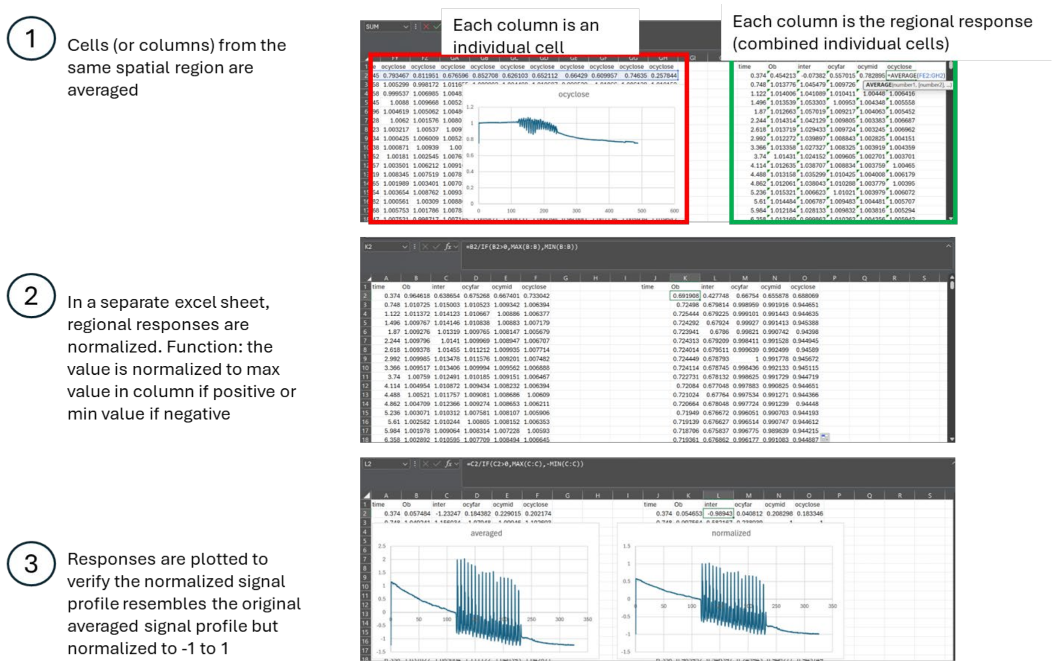

Figure S4 Process workflow used to characterize calcium signaling activity within chips for proximal, medial and distal regions within chamber 2 and cells in chamber 1. This involves averaging and normalizing the responses of individual cells.

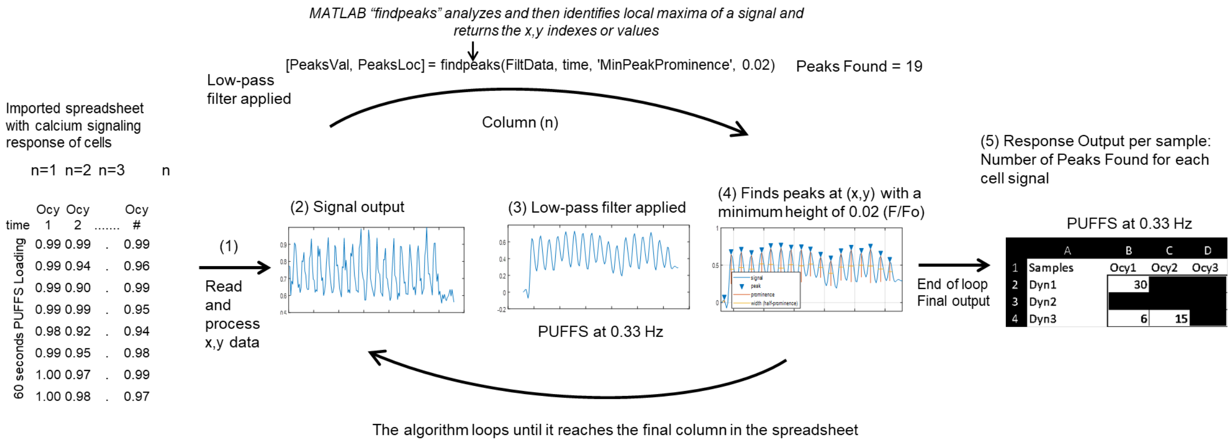

Figure S5 The MATLAB *findpeaks* function (*DistSigPlot_FN.m*) was used to identify the number of peaks within a signal during the application of PUFFS (60s. 0.33Hz). Cells were identified and characterized as responsive if generated signal met the 0.33 Hz frequency threshold (~19 to 33 peaks). The amplitude or minimum peak prominence threshold is 0.02 (F/F0) for detectable peaks. A low-pass filter was applied to reduce noise and potentially prevent false peak detection.

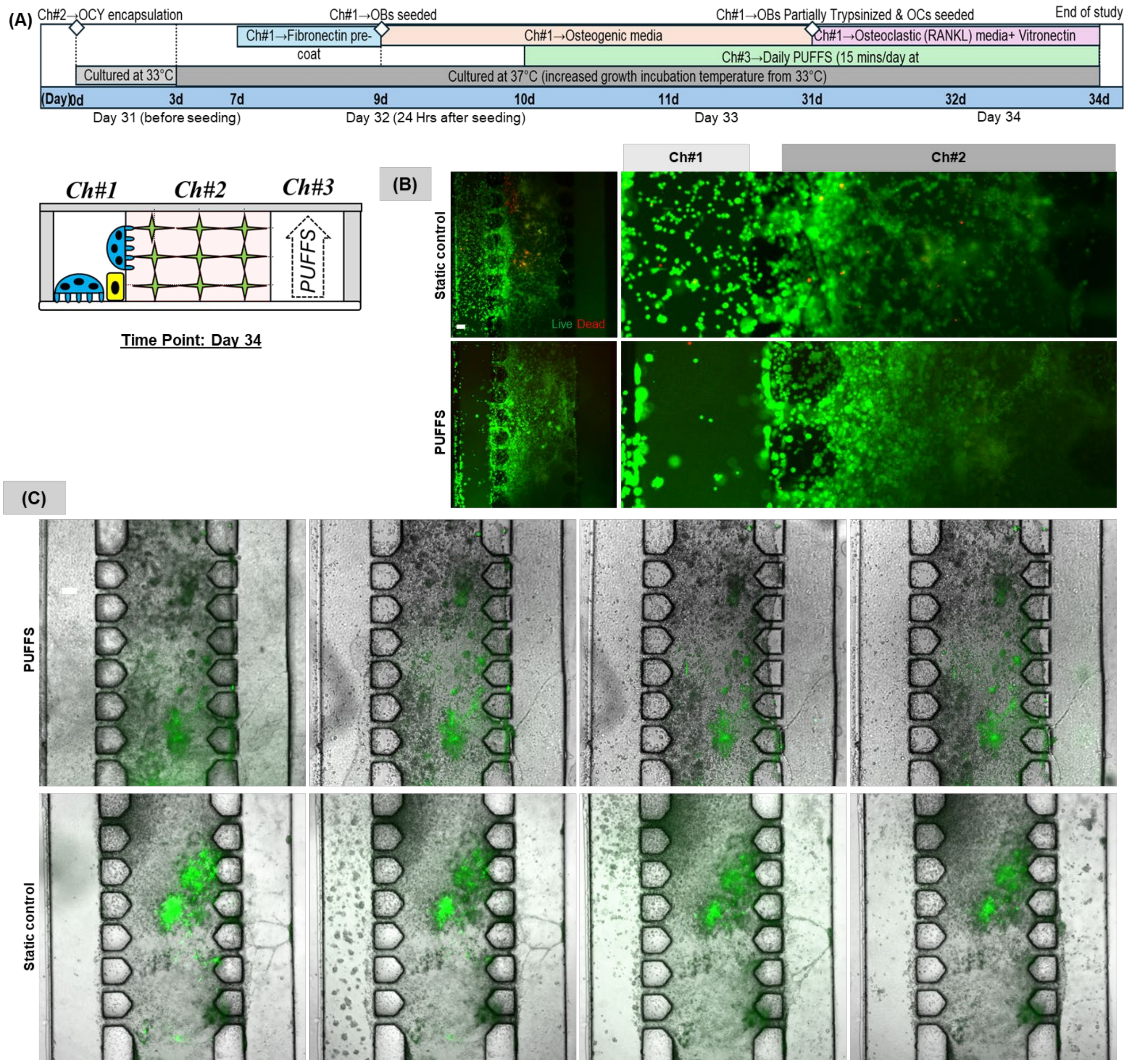

Figure S6 Process flow for development of cultures (A). Composite brightfield and fluorescent (B) up to day 34 images for Static (No stimuli) and Dynamic (daily PUFFS) Triculture cultures at 4mg/mL (top panel). Representative shows (D) fluorescent images of live (green) and dead (red) cells.

Table S2 Oligonucleotide Primers and Relative fold-change of gene expression by RT-qPCR for tricultures (Static no stimuli vs. Dynamic (daily PUFFS))

Gene	Symbol	Static v. Dynamic Fold Change ($2^{-\Delta\Delta CT}$)	Relevance	Primer sequence
Integrin beta-3	*ITGB3*	2.13	Resorption-based adhesion	F:5'-CCACACGAGGCGTGAACTC-3'<br>R: 5'-CTTCAGGTTACATCGGGGTGA-3'
Tartrate-resistant acid phosphatase	*TRAP*	0.66	Facilitates osteoclast adhesion to resorb by downregulating OPN	F: 5'-CACTCCCACCCTGAGATTTGT-3'<br>R: 5'-CATCGTCTGCACGGTTCTG-3'
Receptor Activator of Nuclear Factor-κB	*RANK*	41.87	Role in remodeling and osteoclast differentiation	F: 5'-GGACGGTGTTGCAGCAGAT-3'<br>R: 5'-GCAGTCTGAGTTCCAGTGGTA-3'
Nuclear Factor of Activated T Cells 1	*NFATc1*	6.36	Regulates pre-osteoclast fusion into larger multinucleated osteoclasts	F: 5'-GACCCGGAGTTCGACTTCG-3'<br>R: 5'-TGACACTAGGGGACACATAACTG-3'
Green Fluorescent Protein	*GFP*	0.00089	Identify Ocy454 cells	F: 5'-ACTACAACAGCCACAACGTCTATATCA-3'<br>R: 5'-GGCGGATCTTGAAGTTCACC-3
	GAPDH		HSK	F: 5'-AGGTCGGTGTGAACGGATTTG-3'<br>R: 5'-TGTAGACCATGTAGTTGAGGTCA-3'
	HPRT1		HSK	F: 5'-TCAGTCAACGGGGGACATAAA-3'<br>R: 5'-GGGGCTGTACTGCTTAACCAG-3'
	Actin-β		HSK	F: 5'-GGCTGTATTCCCCTCCATCG-3'<br>R: 5'-CCAGTTGGTAACAATGCCATGT-3'

Housekeeping Genes (HSK) = Average CT value GAPDH, HPRT1, BetaActin

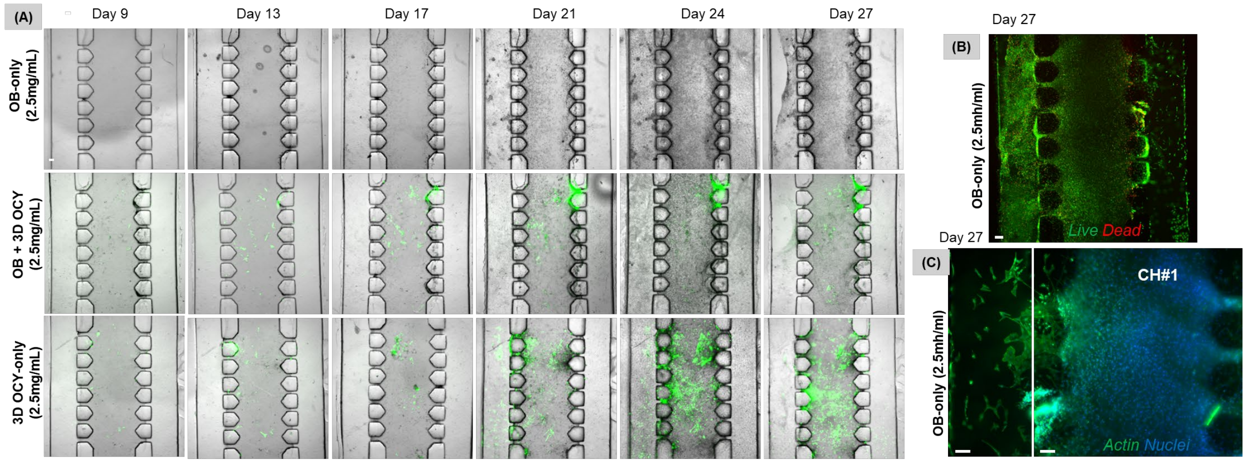

Figure S7 (A) Composite brightfield and fluorescent images showing expression of DMP-1 (GFP) in chamber 2 of the chip for 2.5mg/ml collagen concentration. Scale bar = 100 µm. Cell viability (B) and cell morphology (C) of OB-only chip showing migration of cells from chamber 1 into 2.5mg/ml collagen gel (B) live =green; dead= red. (C) Green=f-actin; blue=nuclei.

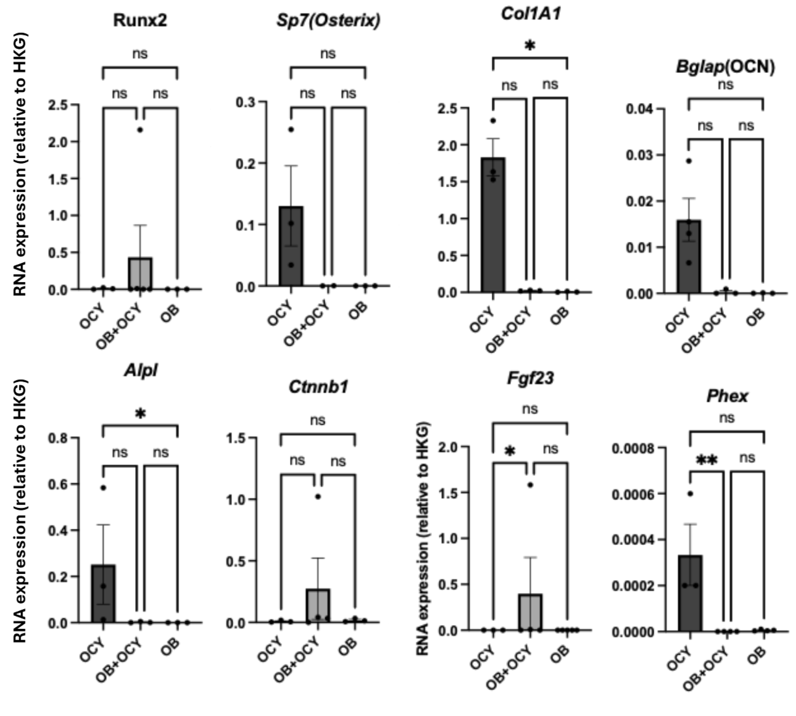

Figure S8. Expression of Phenotypic marker transcripts by RT-qPCR.

We assayed the expression of a number of genes noted in the literature as markers of the osteoblast and/or osteocyte phenotype but acknowledge that inclusion of dexamethasone in the osteogenic differentiation medium used to in mono-culture of the MC3T3-E1.4 pre-osteoblasts and co-cultures with OCY454 cells is a major confounder in these experiments. In early differentiation phases, dexamethasone can transiently support osteogenic programming and increase expression of selected osteogenic markers, but with continued exposure it inhibits terminal osteoblast differentiation, suppresses matrix gene expression via Runx2/Wnt inhibition, and increases Fgf23 while perturbing Dmp1 and Phex, thereby distorting the apparent transcriptomic trajectory from osteoblast to osteocyte.[1] Consequently, differences between murine osteoblast monocultures and osteoblast/osteocyte co-cultures in this Dex-containing medium may reflect the intersection of glucocorticoid pharmacologic effects on bone rather than intrinsic osteogenic or mechanotransductive programs alone.[2-3] A summary of the function of each of these genes, expected level of expression osteoblastic differentiation and osteocytic maturation, as well as the effect of dexamethasone treatment on their expression follows:

Runt-related transcription factor 2 (*Runx2)* and Osterix (*Sp7*) are considered the master transcription factors for initiating osteoblast lineage commitment, osteoblastic differentiation and bone matrix synthesis.[4-5] While *Sp7* is a downstream target of *Runx2,* expression of both genes expression peaks early osteoblast differentiation, gradually

falling to undetectable levels in mature osteocytes. Osteoprogenitors and committed osteoblasts exposed to mechanical loading show upregulation and/or stabilization of Runx2**,** in turn upregulating *Sp7*, to recruit new osteoblasts and prolong the the osteoblast phenotype by delaying osteocytic differentiation via Wnt1-dependent Β-catenin pathways.[6-9] Interestingly, our experiments showed Runx2 expressed only in the co-culture condition, while Sp7 was detected only in the osteocyte monoculture. Like ALPL, this result may reflect the use of dexamethasone in thec osteogenic induction media in the OB and OB+OCY conditions, which reduces Runx2 expression via miR-145a-3p, suppressing downstream osteogenic genes like *Sp7.*[1, 4]

The alpha-1 chain of type I collagen gene (*Col1a1*) codes for Type I collagen, the principal organic matrix component synthesized by osteoblasts.[10] It is highly expressed in early and mid-stage osteoblasts during matrix synthesis, and may be upregulated by mechanical strain/compression. As the cells mature however, Col1a1 expression falls markedly as cells embed themselves in their matrix and become osteocytes. In our experiments, Col1a1 was detected only at low levels in the OB and OB+OCy chips, and was highly expressed in the Ocy mono culture, likely due to chronic exposure to Dexamethasone, which which limits matrix deposition and osteoblast-to-osteocyte embedding.[3, 11]

Osteocalcin (*Bglap*) is a non-collagenous bone matrix protein associated with mineralization and bone turnover. While being expressed only at low levels in early osteoblasts and osteocytes, Bglap is considered a classic late osteoblast marker, with mechanical loading generally demonstrated to enhance expression in late-osteoblasts. Overall, Bglap expression was very low in our experiments; it was not detected in the osteoblast monoculture, barely detected in the co-culture, with highest expression in the osteocyte monoculture. As a Bglap is regulated by Runx2 and Sp7, these results may again reflect the inclusion of Dexamethasone in the induction media.[1, 3]

While expressed in several organs and tissues, in bone, tissue-nonspecific alkaline phosphatase (*ALPL*) is essential to matrix mineralization, hydrolysing pyrophosphate to provide ionic phosphate ions for incorporation into hydroxyapatite. Osteoblasts are understood to express high levels of ALPL during osteoid secretion, with levels generally falling as they become entombed in matrix and mature into osteocytes. In our experiment ALPL was detected at the highest level in monocultured Ocy, but was not detected in either mono cultured OB or the OB+Ocy co-culture. While unexpected, this may reflect that the OB and OB+OCy were cultured in osteogenic induction media that contained Dexamethasone, and which has been reported to suppress ALPL expression in the murine osteoblasts.[12]

β-catenin (*Ctnnb1*) is the central effector of canonical Wnt signaling, which drives osteoblast differentiation, survival and osteocyte mechanotransduction.[13] β-catenin is

expressed at moderate levels in osteoblasts and osteocytes, with activity increased by Wnt ligands and mechanical loading to promote osteogenesis and the osteoblast-to-osteocyte transition. In our experiments, *Ctnnb1* was barely detectable in either monoculture condition, with highest levels in the OB+OCy co-cultures. This result may reflect secretion of Wnt1 by the OCy, being received by the OB to induce *Ctnnb1* expression in the co-culture configuration. However, the low level of expression overall may be a consequence of chronic Dexamethasone exposure, which reduces β-catenin signaling (e.g. via induction of Wnt antagonists) and thus counteracts mechanically and Wnt-driven bone-forming responses.

Fibroblastic Growth factor 23 (*Fgf23)* is a phosphaturic hormone produced predominantly by osteocytes that decreases renal phosphate reabsorption and 1,25$(OH)_2$D synthesis. Its expression is minimal in osteoblasts, but increases to high levels in a subset of mature osteocytes. Our experiments showed barely detectable levels of FGF-23 expression in both OB and OCy monocultures, and moderate expression in the co-culture. *FGF-23*, is suppressed by mechanical loading, and is robustly induced by glucocorticoids (e.g dexamethasone), shifting the Dmp1–Phex–Fgf23 axis toward a phosphaturic, mineralization-inhibiting profile.

The gene coding for Phosphate regulating endopeptidase X-linked protein (*Phex*) is an endopeptidase expressed in bone that regulates matrix peptides and restrains excess Fgf23 secretion, thereby contributing to normal phosphate and mineral homeostasis. Expression is low in early osteoblasts, moderate in late osteoblasts, and high in early and mature osteocytes. Mechanical loading tends to maintain or modestly increase Phex expression, favoring a Dmp1/Phex-high, Fgf23-low phenotype. In our experiments, Phex expression was undetectable in OB mono cultures and OB+OCY co-cultures, and detected at only at low levels in the osteocyte monocultures. While dexamethasone exposure has been reported to increase Phex mRNA in osteocytes, however this study was conducted in the context of osteocyte injury, which may limit its FGF-23-limiting effect due to simultaneous FGF-23 induction.[2]

Table S3: Comparison of this work with similar in vitro culture models in the field.

	Culture type	Types of *in vitro* models	Compatibility with real-time signaling	Ability to apply (cyclic) mechanical stimuli
Current work	OCY-OB-OC (OCY454)	3D culture in microfluidic chip	✓	✓
Merife et al., 2025[14]	OCY-only (MLO-Y4)	3D culture in microfluidic chip	✓	✓
Zhang et al., 2022 [15]	OCY-only (MLO-Y4)	2D culture + GelMA hydrogel	✓	✓
Zhang et al., 2022 [16]	hMSC	3D bioprinting		✓
Yvanoff et al., 2022[17]	OCY-OB	2D cell patterns + microfluidic chip	✓	✓
Wilmoth et al., 2020[18]	OCY-only	3D printed composite + cell-laden gel		✓
Matsuzaka et al., 2021[19]	OCY-OB	2D culture + soluble exchange of media between chambers	✓	✓
Middleton et al., 2017[20]	OCY-OC	Microfluidic chip	✓	✓
Bernhadt et al., 2021[21]	OCY-OB-OC	Transwell inserts		
Wirsig et al., 2022[22]	OCY-OB-OC	Transwell inserts		
Woo et al., 2025[23]	OCY-OB-OC	Custom PDMS Transwell inserts		
Truesdell et al., 2019[24]	OCY-only	Flow chamber + 2D culture	✓	✓

Movie 1 Caption. 3D reconstruction of OCY454 networks within 3D collagen gel in chamber 2 of OB+OCY co-cultured chip on Day 31 using Image J volume viewer.

References.

1. Wu, H.; Liao, X.; Wu, T.; Xie, B.; Ding, S.; Chen, Y.; Song, L.; Wei, B., Mechanism of Mir-145a-3p/Runx2 Pathway in Dexamethasone Impairment of Mc3t3-E1 Osteogenic Capacity in Mice. *PloS one* **2024,** *19* (11), e0309951.
2. Rial-Pensado, E.; Canaple, L.; Guyot, R.; Clemmensen, C.; Wiersema, J.; Wu, S.; Richard, S.; Boelen, A.; Müller, T. D.; López, M., Neuronal Blockade of Thyroid Hormone Signaling Increases Sensitivity to Diet-Induced Obesity in Adult Male Mice. *Endocrinology* **2023,** *164* (4), bqad034.
3. Umrath, F.; Pfeifer, A.; Cen, W.; Danalache, M.; Reinert, S.; Alexander, D.; Naros, A., How Osteogenic Is Dexamethasone?—Effect of the Corticosteroid on the Osteogenesis, Extracellular Matrix, and Secretion of Osteoclastogenic Factors of Jaw Periosteum-Derived Mesenchymal Stem/Stromal Cells. *Frontiers in Cell and Developmental Biology* **2022,** *10*, 953516.
4. Rashid, H.; Ma, C.; Chen, H.; Wang, H.; Hassan, M. Q.; Sinha, K.; De Crombrugghe, B.; Javed, A., Sp7 and Runx2 Molecular Complex Synergistically Regulate Expression of Target Genes. *Connective tissue research* **2014,** *55* (sup1), 83-87.
5. St. John, H. C.; Bishop, K. A.; Meyer, M. B.; Benkusky, N. A.; Leng, N.; Kendziorski, C.; Bonewald, L. F.; Pike, J. W., The Osteoblast to Osteocyte Transition: Epigenetic Changes and Response to the Vitamin D3 Hormone. *Molecular endocrinology* **2014,** *28* (7), 1150-1165.
6. Ahmad, M.; Haffner-Luntzer, M.; Schoppa, A.; Najafova, Z.; Lukic, T.; Yorgan, T. A.; Amling, M.; Schinke, T.; Ignatius, A., Mechanical Induction of Osteoanabolic Wnt1 Promotes Osteoblast Differentiation Via Plat. *The FASEB Journal* **2024,** *38* (4), e23489.
7. Liu, P.; Tu, J.; Wang, W.; Li, Z.; Li, Y.; Yu, X.; Zhang, Z., Effects of Mechanical Stress Stimulation on Function and Expression Mechanism of Osteoblasts. *Frontiers in Bioengineering and Biotechnology* **2022,** *10*, 830722.
8. Deng, H.; Wan, D., Mechanical Loading Regulates Osteogenic Differentiation and Bone Formation by Modulating Non-Coding Rnas. *PeerJ* **2025,** *13*, e19310.
9. Yoshida, C. A.; Komori, H.; Maruyama, Z.; Miyazaki, T.; Kawasaki, K.; Furuichi, T.; Fukuyama, R.; Mori, M.; Yamana, K.; Nakamura, K., Sp7 Inhibits Osteoblast Differentiation at a Late Stage in Mice. *PloS one* **2012,** *7* (3), e32364.
10. Prockop, D. J.; Kivirikko, K. I., Collagens: Molecular Biology, Diseases, and Potentials for Therapy. *Annual review of biochemistry* **1995,** *64*, 403-434.
11. Coxon, F. P.; Thompson, K.; Roelofs, A. J.; Ebetino, F. H.; Rogers, M. J., Visualizing Mineral Binding and Uptake of Bisphosphonate by Osteoclasts and Non-Resorbing Cells. *Bone* **2008,** *42* (5), 848-860.
12. Orriss, I. R.; Hajjawi, M. O.; Huesa, C.; MacRae, V. E.; Arnett, T. R., Optimisation of the Differing Conditions Required for Bone Formation in Vitro by Primary Osteoblasts from Mice and Rats. *International journal of molecular medicine* **2014,** *34* (5), 1201-1208.
13. Gomez-Ospina, N.; Tsuruta, F.; Barreto-Chang, O.; Hu, L.; Dolmetsch, R., The C Terminus of the L-Type Voltage-Gated Calcium Channel Cav1. 2 Encodes a Transcription Factor. *Cell* **2006,** *127* (3), 591-606.

14. Merife, A.-B.; Poudel, A.; Polshikova, A.; Geffert, Z. J.; Horton, J. A.; Hasan Akash, M. M.; Pandey, A.; Basu, S.; Fougnier, D.; Soman, P., 3d Osteocyte Networks under Pulsatile Unidirectional Fluid Flow Stimuli (Puffs). *ACS Biomaterials Science & Engineering* **2025,** *11* (10), 6216-6233.
15. Zhang, K.; Ogando, C.; Filip, A.; Zhang, T.; Horton, J. A.; Soman, P., In Vitro Model to Study Confined Osteocyte Networks Exposed to Flow-Induced Mechanical Stimuli. *Biomedical Materials* **2022,** *17* (6), 065027.
16. Zhang, J.; Griesbach, J.; Ganeyev, M.; Zehnder, A.-K.; Zeng, P.; Schädli, G. N.; de Leeuw, A.; Lai, Y.; Rubert, M.; Müller, R., Long-Term Mechanical Loading Is Required for the Formation of 3d Bioprinted Functional Osteocyte Bone Organoids. *Biofabrication* **2022,** *14* (3), 035018.
17. Yvanoff, C.; Willaert, R. G., Development of Bone Cell Microarrays in Microfluidic Chips for Studying Osteocyte–Osteoblast Communication under Fluid Flow Mechanical Loading. *Biofabrication* **2022,** *14* (2), 025014.
18. Wilmoth, R.; Ferguson, V.; Bryant, S., A 3d, Dynamically Loaded Hydrogel Model of the Osteochondral Unit to Study Osteocyte Mechanobiology, Adv. Healthcare Mater. 9 (2020) 2001226.
19. Matsuzaka, T.; Matsugaki, A.; Nakano, T., Control of Osteoblast Arrangement by Osteocyte Mechanoresponse through Prostaglandin E2 Signaling under Oscillatory Fluid Flow Stimuli. *Biomaterials* **2021,** *279*, 121203.
20. Middleton, K.; Al-Dujaili, S.; Mei, X.; Günther, A.; You, L., Microfluidic Co-Culture Platform for Investigating Osteocyte-Osteoclast Signalling during Fluid Shear Stress Mechanostimulation. *Journal of biomechanics* **2017,** *59*, 35-42.
21. Bernhardt, A.; Skottke, J.; von Witzleben, M.; Gelinsky, M., Triple Culture of Primary Human Osteoblasts, Osteoclasts and Osteocytes as an in Vitro Bone Model. *International journal of molecular sciences* **2021,** *22* (14), 7316.
22. Wirsig, K.; Kilian, D.; von Witzleben, M.; Gelinsky, M.; Bernhardt, A., Impact of Sr2+ and Hypoxia on 3d Triple Cultures of Primary Human Osteoblasts, Osteocytes and Osteoclasts. *European Journal of Cell Biology* **2022,** *101* (3), 151256.
23. Woo, S.-M.; Paek, K.; Yoon, Y. M.; Kim, H.; Park, S. I.; Kim, J. A., Development of a Bmu-on-a-Chip Model Based on Spatiotemporal Regulation of Cellular Interactions in the Bone Remodeling Cycle. *Materials Today Bio* **2025,** *32*, 101658.
24. Truesdell, S.; George, E.; Seno, C.; Saunders, M., 3d Printed Loading Device for Inducing Cellular Mechanotransduction Via Matrix Deformation. *Experimental Mechanics* **2019,** *59* (8), 1223-1232.
